# Citation and policy influence of research using demographic and health survey data: a bibliometric analysis

**DOI:** 10.1186/s12961-026-01487-0

**Published:** 2026-05-14

**Authors:** Evans Omondi, Eliud Wekesa, Symon M. Kariuki, Rachel Odhiambo, Daniel Osuka, Soumaila Ouedraogo, Patricia Kitsao-Wekulo, Agnes Kiragga, Catherine Kyobutungi

**Affiliations:** 1 African Population and Health Research Center, Nairobi, Kenya; 2 Institute of Mathematical Sciences, Strathmore University, Nairobi, Kenya; 3 Department of Sociology and Community Development, South Eastern Kenya University, Kitui, Kenya

**Keywords:** Academic impact, Demographic and health survey, Policy influence, Public health, Research evaluation, Societal impact

## Abstract

**Background:**

While citation counts are critical metrics for scholarly impact of research articles, they cannot objectively measure other impacts of research to society. Research articles have broader impact beyond academic, including informing health policy-making, planning and practice, but this utility has not been systematically examined for the Demographic and Health Survey (DHS)-based publications. This paper examines both the academic and policy-related impacts of DHS-based articles.

**Methods:**

A systematic search was conducted in PubMed, Scopus, Web of Science, Cumulative Index to Nursing and Allied Health Literature (CINAHL), Wiley Online Library and Dimensions and grey literature (theses and dissertations) to identify DHS-based publications since 1984. Academic impact was assessed through journal destinations, scope, accessibility and citation counts. To assess policy impact, this study utilizes the Overton database to identify citations of scientific research in policy documents.

**Results:**

Citation to DHS-based publications have increased over the last four decades, contributing significantly to the public health evidence that has been utilized for academic and policy in low- and middle-income countries (LMICs). Multidisciplinary and open-access journals such as *PLOS One* have predominantly published DHS-citation related research, often led by researchers from high-income countries (HICs). While open-access has improved accessibility for LMIC-led research, citation impact is skewed towards HIC-led studies, suggesting inequities in the citation impact landscape. The steady increase in both scholarly and policy citations indicates that DHS-based research is an important resource for academic and global health policy-making.

**Conclusions:**

DHS-based evidence plays a critical role in both academic and policy spheres. Its consistent citation growth demonstrates the scientific value of open, standardized, nationally representative data and its citation growth in policy documents underscores the need for continued investment in the programme to support evidence-based decision-making in LMICs.

## Introduction

The pursuit of scientific knowledge is fundamentally driven by the desire to understand the world and to apply that to improve human well-being. In public health, this translational imperative is paramount; research is a vital tool for crafting effective policies, planning equitable health systems and implementing life-saving interventions [1, 2]. It becomes clear that research is valuable not only for its academic impact but also for wider impact on society. Yet for decades, the gold standard for measuring the value of scholarly work has been the citation count, a metric that quantifies academic influence within the scientific community [3, 4]. Citations reflect contribution to scientific advancement through theory development and methodological innovation [5]. While citations offer a tangible, albeit imperfect, measure of scholarly discourse and impact, they often fail to capture the broader societal value of research, especially in applied disciplines such as public health [6–8]. Although citation metrics provide useful information on scientific productivity patterns, they rarely reveal whether research evidence informs public health policies, programs and planning. This gap between academic acclaim and tangible societal benefit represents a critical blind spot in our understanding of how research truly shapes policy and practice.

While metrics for measuring academic impact, particularly citation-based indicators, are well established and widely standardized, approaches for assessing the broader societal impact of research have emerged more recently and remain conceptually and methodologically diverse and under active development [7, 9–15]. One of the new ways of measuring societal impact of research is Altmetrics, short for alternative metrics, which tracks social media and other online engagements with scholarly work rather than traditional citations [16]. Although initially promising, recent studies have highlighted the limitations of Altmetrics. Altmetrics can be limited by the technological ecosystem and not being direct measures of social impact but indicators of interaction and spread of research beyond academic audience [16]. Consequently, the integration of scientific articles into policy documents has been suggested as a better and more powerful indicator of the impact of research on society [17, 18]. The citation of academic articles in policy documents increases both the credibility of the authors cited and the policy documents citing them, which demonstrates the nexus between academic research and policy-making [18].

The emergence of the Overton database offers a novel opportunity to systematically analyse the policy impact of scientific research by tracing citations of scholarly outputs within policy documents [19]. Recent research impact assessments have increasingly leveraged Overton data to examine the translation of research into policy across thematic areas such as climate change, public health and institutional research performance [19–22]. The Overton database, a large comprehensive repository of policy documents, was released in 2019, featuring citation linkages to research papers referenced within those policy documents. Overton defines policy documents broadly as materials produced for or by policy-makers, including outputs from governments, think tanks, nongovernmental organizations (NGOs) and intergovernmental organizations (IGOs) [19, 22]. Beyond basic bibliographic details such as publication titles, dates and topics, the database uses automated text-mining techniques to extract citation relationships. These include links between policy documents and scholarly literature, as well as citations within the policy corpus itself. Scholarly references in Overton are identified through digital object identifiers (DOIs), enabling standardized and reliable connections between scientific research and policy outputs [19]. The database is updated weekly and covers many countries and populations, with the exception of the mainland China [23]. As of December 2020, the database contained nearly 800 000 policy documents in 66 languages from 168 countries, sourced from more than 1250 policy-issuing organizations. Its extensive coverage includes diverse document types such as government guidelines, policy briefs, think tank reports and technical working papers, drawn from more than 1000 institutions across more than 180 countries [22, 23].

Nowhere is this distinction between academic and societal impact more salient than in low- and middle-income countries (LMICs), where resource constraints necessitate that evidence must be not only scientifically robust but also directly actionable [24]. For more than four decades, the Demographic and Health Surveys (DHS) programme has served as a cornerstone of public health evidence generation in these settings. By providing high-quality, nationally representative and internationally comparable data across numerous health and demographic indicators, the DHS programme has equipped researchers and policy-makers with policy-relevant evidence on population health and development trends [25, 26]. DHS surveys collect detailed data on fertility and family planning, maternal and child health, nutrition, mortality, HIV and other infectious diseases, gender-based violence, health service utilization, household characteristics and socioeconomic inequalities. These data are typically analysed to address a wide range of research questions, including monitoring trends and disparities in health outcomes, identifying determinants of adverse health events, evaluating the coverage and equity of key interventions and assessing progress towards global and national development targets such as the Sustainable Development Goals. The resulting corpus of thousands of peer-reviewed publications analysing DHS data constitutes a vast evidence base informing issues from maternal and child mortality to nutrition, fertility and infectious diseases, as well as cross-cutting themes such as geographic, socioeconomic and gender inequalities in health outcomes [27]. This evidence has the potential to inform diverse policy questions, including priority-setting for national health strategies, allocation of resources across regions and population groups, design of targeted interventions for vulnerable populations and evaluation of programme performance and accountability frameworks. The academic impact of DHS-based publications is evident in their proliferation across scholarly journals and their accumulation of citations, contributing significantly to scientific careers and academic discourse [28, 29]. However, the ultimate test of this research lies in its broader utility: whether this extensive body of DHS-based evidence has successfully informed global and national health policy agendas, shaped strategic planning processes or guided implementation and monitoring of interventions on the ground.

**Fig. 1:**
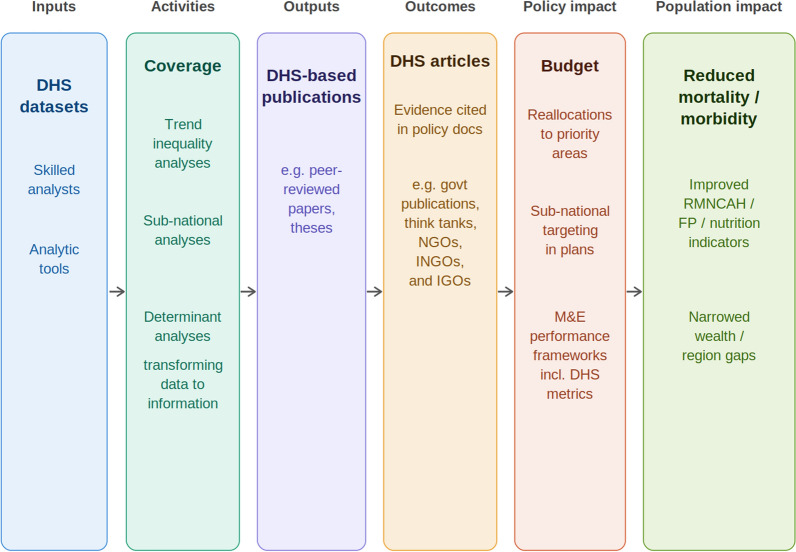
Theory of change (DHS research $\rightarrow$ policy impact)

Citing DHS-based research in policy documents can achieve policy impact through which the increased accessibility of the research evidence is translated into actionable priorities for decision-makers, such as identifying high-burden populations or geographic areas, determining priority health interventions, informing the allocation of limited resources, setting measurable targets for national strategies and strengthening monitoring and evaluation frameworks for accountability. DHS-based research captures the scale, trends and reveals inequalities and regional and social disparities in health and development indicators [25]. To have policy impact, these data should be transformed into information, which must be accessible to policy-makers. When this evidence is cited in policy documents, it enhances its credibility, helps define key priorities (agenda setting), informs more targeted interventions, supports advocacy and improves monitoring and evaluation for accountability [18, 21, 30]. DHS research-citing documents contribute to the policy evidence base by informing agenda setting, shaping policy discourse and supporting the formulation, review or justification of national and subnational strategies, resource-targeting approaches and programmatic priorities. Rather than representing direct policy decisions in all cases, many policy documents indexed in Overton reflect the use of scientific evidence to underpin policy analysis, guidance, advocacy and deliberative processes involved in policy-making [4, 15, 19]. As such, citations of DHS-based research in Overton provide a traceable and systematic indicator of the integration of population-based evidence into policy-relevant documents, offering insight into the pathways through which research may contribute to policy formulation and implementation, albeit without implying direct attribution to final policy decisions [15]. The theory of change depicting DHS based research-to-policy impact pathways is shown in Fig. 1.

While anecdotally praised, the systematic examination of DHS literature’s policy impact remains a conspicuously understudied area, supporting a narrative that is rich in academic metrics but poor in documented real-world effect. This work seeks to bridge this critical knowledge gap by conducting a dual assessment of the policy and citation wielded by DHS-based research. We move beyond traditional bibliometrics to provide a comprehensive analysis of both academic and policy impact. Our study systematically identifies the landscape of DHS publications, examining their journal destinations, accessibility and academic and policy citation metrics. By mapping citation trends and patterns and references in policy documents, the study aims to provide a comprehensive overview of how research contributes to both scientific advancement and policy development irrespective of public health discipline. Such evidence is essential for strengthening research–policy linkages, informing institutional strategies and guiding future investment in impactful health research. The work focuses on the DHS programme because it is a standardized, nationally representative and widely trusted source of high quality and reliable population health data in LMICs. The DHS programme was established not only to generate high-quality demographic and health indicators but also to support evidence-informed decision-making by governments and development partners through the systematic production, dissemination and accessibility of standardized population-based data [30]. Evidence of uptake by these actors can be observed through citations of DHS-based research and reports within policy-relevant documents produced by governments, intergovernmental organizations and development partners, as captured in policy citation databases such as Overton. While these citations do not necessarily represent final policy decisions, they demonstrate that DHS-derived evidence is being drawn upon to inform policy analysis, programme design, monitoring frameworks and strategic planning processes within national and international policy arenas. This therefore offers an important opportunity to assess how the evidence generated from DHS data contributes to scholarly knowledge and influences policy.

## Materials and methods

This study employed a comprehensive mixed-methods systematic review, combining a systematic bibliometric review with a novel policy document analysis to assess both the academic and policy impact of publications derived from the Demographic and Health Surveys (DHS) programme.

### Study design and data sources

This study was designed as a comprehensive mixed-methods systematic review analysis to evaluate both the academic and policy impact of research publications utilizing DHS programme data. The methodology integrated a systematic bibliometric review, which quantitatively assesses scholarly output and impact, with a novel policy document analysis, which qualitatively and quantitatively traces the uptake of research into the policy arena. To construct the most complete corpus of DHS-based literature possible, our search extended across six major electronic bibliographic databases renowned for their coverage of the biomedical, public health and social sciences fields: PubMed, Scopus, Web of Science, CINAHL, Wiley Online Library and Dimensions. Recognizing that significant scholarly work, particularly from LMICs, is often disseminated through academic theses, we also conducted a systematic search of grey literature sources, including ProQuest Dissertations and Theses Global and other relevant institutional repositories. The temporal scope of our search was designed to encompass the entire history of the DHS program, from its inception in 1984 through to June 2025, with a final search execution date anticipated in early 2025 to ensure the inclusion of all publications from the 2024 calendar year.

### Search strategy

A systematic and reproducible search strategy was developed to identify all relevant publications with high sensitivity and specificity. The strategy was built around the core concept of “Demographic and Health Surveys”, including its common variants and abbreviations such as “Demographic Health Program” and “Demographic Health Study.” To refine our approach, we first conducted a preliminary scoping search to identify a seed set of known DHS-based articles; the keywords and metadata from these articles were analysed to inform the final search string. We utilized both database-specific controlled vocabularies (for example, Medical Subject Headings [MeSH] in PubMed) and free-text keywords searched in the title and abstract fields to maximize the retrieval of relevant records. The search syntax was adapted to comply with the unique rules and functionalities of each database. The full search strategy for each database is provided in Supplementary Table S1 in the supplementary file.

### Eligibility criteria and study selection

The identification of studies for inclusion was governed by predefined eligibility criteria to ensure the consistency and objectivity of the selection process. The study selection process rigorously adhered to the Preferred Reporting Items for Systematic Reviews and Meta-Analyses (PRISMA) guidelines. All records identified from the database searches were collated and deduplicated using both automated features in reference management software and manual inspection. The subsequent screening was conducted in two distinct phases: first, two independent reviewers screened the titles and abstracts of all records against the eligibility criteria; second, the full texts of all records deemed potentially relevant at the first stage were retrieved and subjected to an in-depth assessment by the same independent reviewers. During the full-text review, we specifically examined the methods section of each publication to confirm explicit use of Demographic and Health Survey (DHS) data, including direct statements indicating DHS as a primary or secondary data source (for example, references to specific DHS survey rounds, country surveys or DHS datasets accessed through the DHS programme). Any discrepancies or conflicts regarding inclusion at either stage were resolved through discussion until consensus was reached or by consultation with a third senior reviewer. We included original quantitative research articles and methodological papers that utilized DHS data as a primary or significant secondary data source for analysis. Publications were excluded if they were editorials, commentaries, letters to the editor or conference abstracts that did not present original empirical findings or if they mentioned the DHS only peripherally without directly utilizing its datasets.

### Data extraction and analysis

A standardized, piloted data extraction form was employed to systematically collect relevant information from each included publication. The extracted data encompassed several key categories: (1) bibliometric metadata, including title, authors, publication year, journal name, journal impact factor (or comparable metric) and the institutional and national affiliation of the first and last authors to analyse geographic leadership and collaboration patterns; (2) accessibility, noting the type of Open Access status (for example, Gold, Green, Hybrid, Closed) to assess barriers to knowledge dissemination; (3) academic impact, operationalized through raw citation counts sourced from Scopus, Web of Science and Dimensions to ensure robustness and with the field-weighted citation impact (FWCI) calculated where possible to allow for cross-disciplinary comparison; and (4) content analysis, including the primary health topic, study objectives and the low- or middle-income country focus of the research. The analysed data were then synthesized using a combination of descriptive statistics to report frequencies, trends and central tendencies, and trend analyses were performed to map the evolution of publication and citation metrics over the four-decade period. The 95% confidence intervals were computed for annual citation outputs.

### Assessment of policy impact

To assess the policy impact of scientific research, this study employed the Overton database as the principal source for identifying citations of academic outputs within policy documents. DHS-based scholarly references indexed in Overton were identified through article digital object identifiers (DOIs). To verify that the DOIs identified by Overton as references within the indexed policy documents were valid, we cross-checked all cited DOIs against the search databases: PubMed, Scopus, Web of Science, CINAHL, Wiley Online Library and Dimensions. Only those DOIs that were indexed in at least one of these databases were taken as valid. Citations of DHS-based studies in policy documents provided by Overton are used to quantify the extent to which these research outputs have attracted attention from policy-makers. We analyse how scholarly evidence referenced across Overton-indexed policy documents is distributed with respect to publication year, policy source, geographic origin and policy document topics. Overton is selected owing to its extensive and systematic coverage of diverse policy literature, including governmental guidelines, policy briefs, think tank reports and technical working papers. The database aggregates policy-relevant content from more than 1000 institutions across 182 countries, thereby providing a uniquely global and cross-sectoral view of policy citation activity [21]. This breadth of coverage enhances the capacity to detect explicit references to scientific evidence across a wide range of policy environments and thematic areas. Consequently, the use of Overton strengthens the reliability, representativeness and comprehensiveness of the policy citation analysis, offering a robust empirical basis for evaluating the extent to which scientific research informs policy formulation and decision-making.

### Ethical considerations

As this study utilized publicly available published data and policy documents, it did not require institutional review board (IRB) approval. However, all aspects of the research were conducted with strict adherence to academic integrity and ethical standards for systematic reviews and data analysis.

## Results

### PRISMA search results

**Fig. 2:**
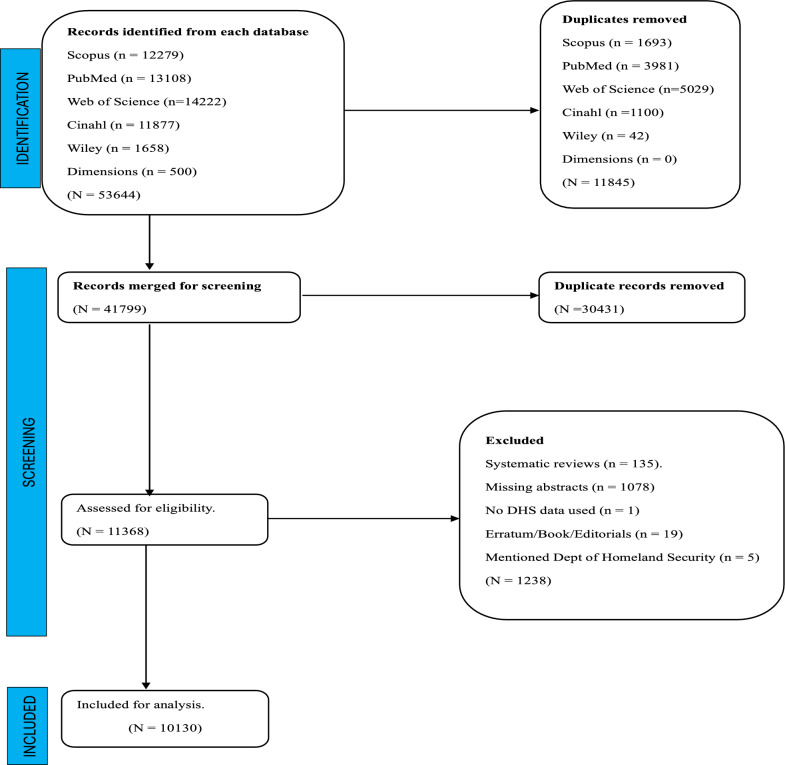
PRISMA flowchart for the study identification, screening process, selection and inclusion in the review

Our systematic search across multiple academic databases initially identified 53,644 potentially relevant publications related to Demographic and Health Surveys (DHS) research. The subsequent screening process was carefully designed to select studies that would best support our comprehensive bibliometric analysis of DHS-based scholarship. The first stage of screening eliminated 11,845 duplicate publications that appeared across each of the databases. We then merged data from all the databases and conducted a thorough review of titles and abstracts, which led to the exclusion of 30,431 articles that appeared in more than one database, ensuring each study was only counted once in our analysis. For citation analysis, priority was given to Scopus entries, as Scopus was selected for its comprehensive coverage of peer-reviewed health literature, consistent citation tracking and compatibility with bibliometric analysis. All citation data presented in this study were extracted from Scopus as of June 2025. At this stage, particular attention was paid to ensuring each potential study contained the necessary metadata for our planned analyses, including complete author affiliation information and citation data. A more rigorous quality assessment resulted in the removal of an additional 1238 studies that lacked methodological rigor, had poorly defined research outcomes or contained incomplete metadata that would have compromised our bibliometric or geospatial mapping. This stringent selection process yielded a final corpus of 10,130 high-quality DHS-based studies that fully met our inclusion criteria. The complete study selection *process is visually summarized*in the Fig. 2, which follows the PRISMA flow diagram format to ensure transparency in our methodology.

### Academic citation trends and patterns

**Fig. 3:**
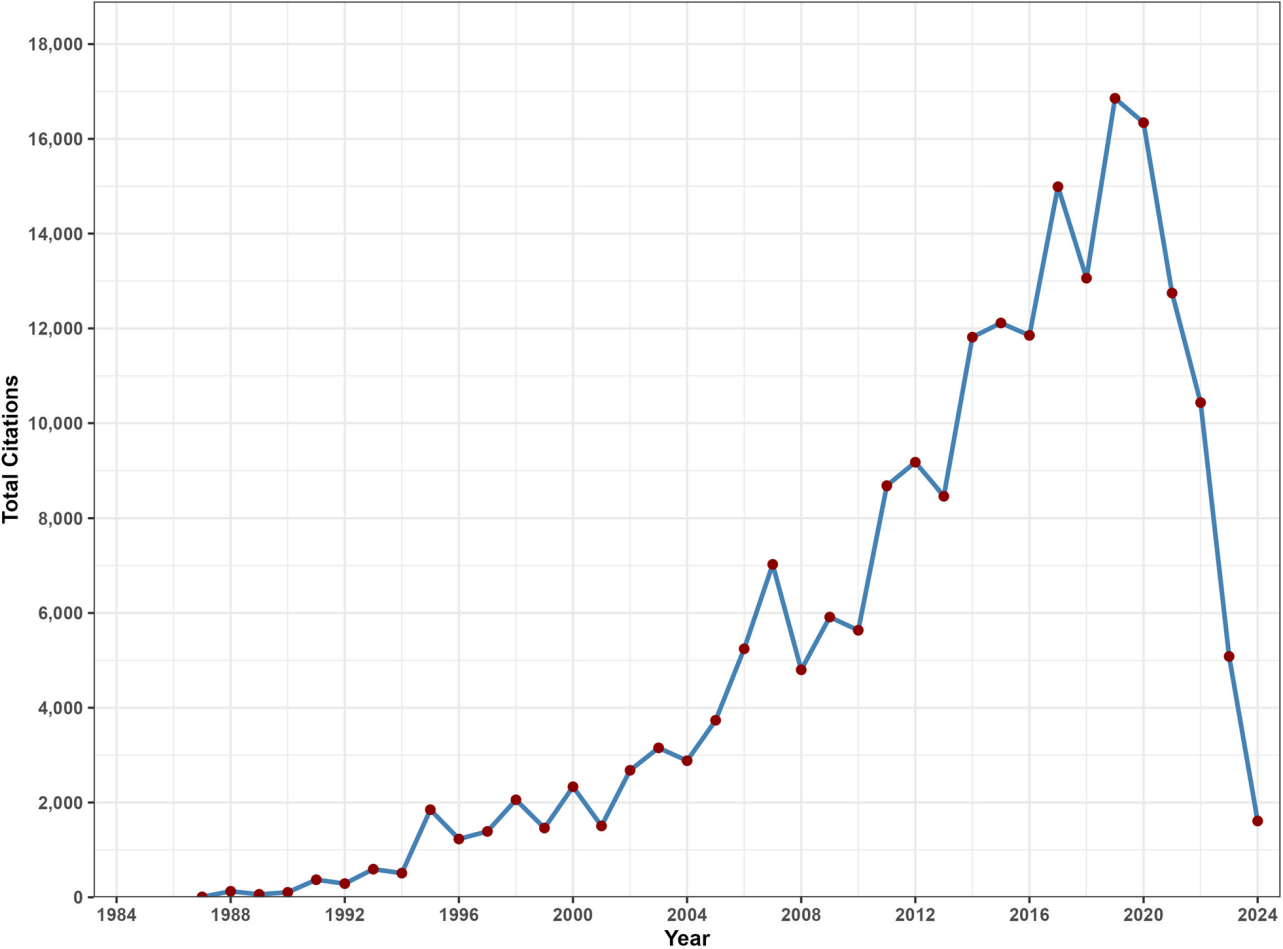
Total citations aggregated by year of the DHS papers. Citation counts were obtained from Scopus and represent cumulative citations per publication year as of June 2025

Figure 3 shows yearly citation trends of DHS-based research from 1984 to 2024. Overall, citations of DHS-related papers increased steadily over the four-decade period. However, citations remained low during the first decade, reflecting limited data and survey coverage and few peer-reviewed articles. A sharp rise began in the 2000s, peaking in the last decade (approximately from 2016), indicating intensified scholarly engagement with DHS findings.

**Fig. 4:**
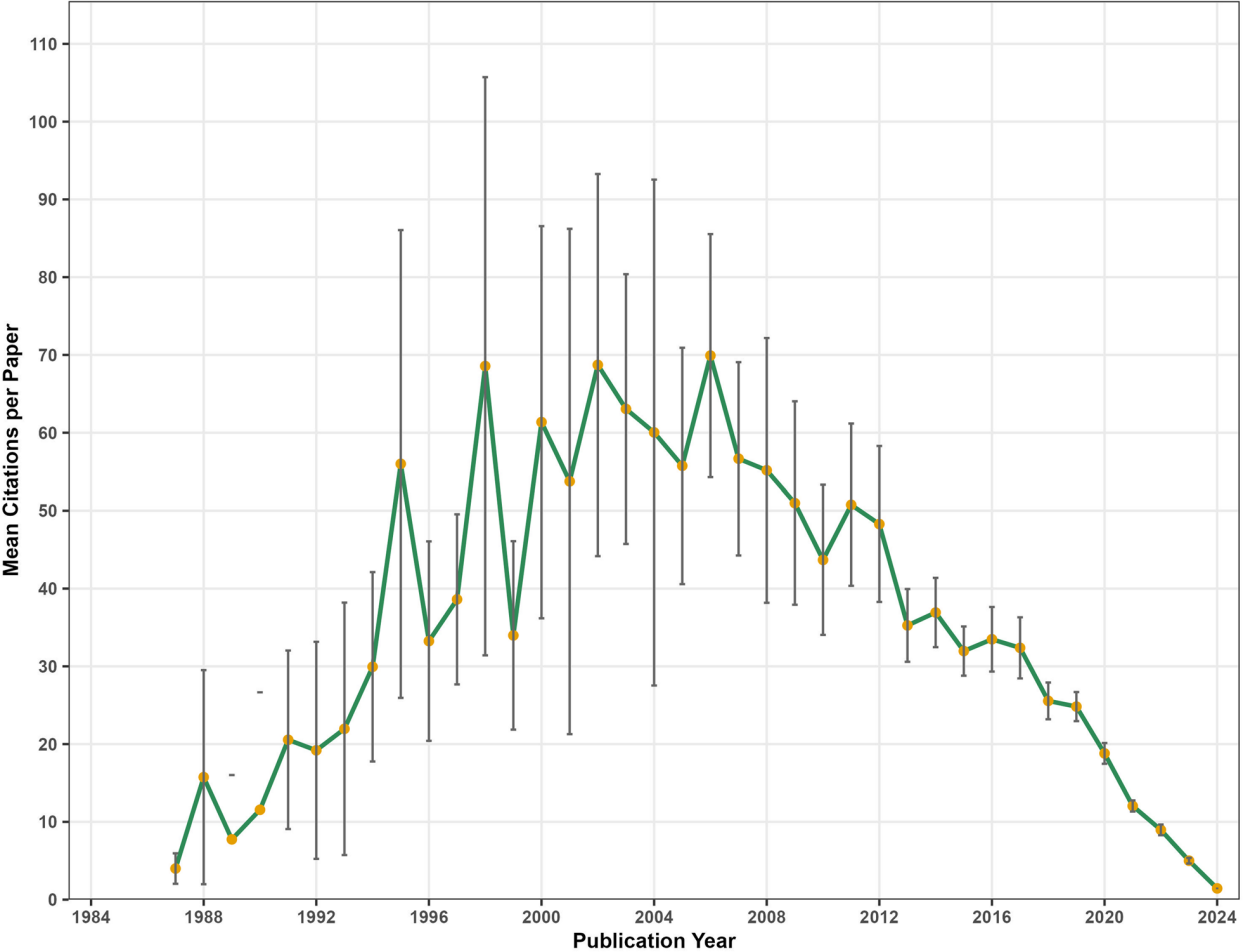
Mean citation rates per DHS paper per year

Fig. 4 presents the mean citation rate per DHS paper per year. The mean citation rate similarly shows an upward trajectory. Earlier publications received fewer citations per paper, while papers published after the late 1990s show higher average citation counts, peaking in the second decade (2000–2010). This pattern reflects both growth in the scientific community using DHS data and the cumulative visibility of DHS research. The rise in mean citations per paper indicates that DHS publications are more frequent and also increasingly influential in academic references.

### Journal analysis

**Fig. 5:**
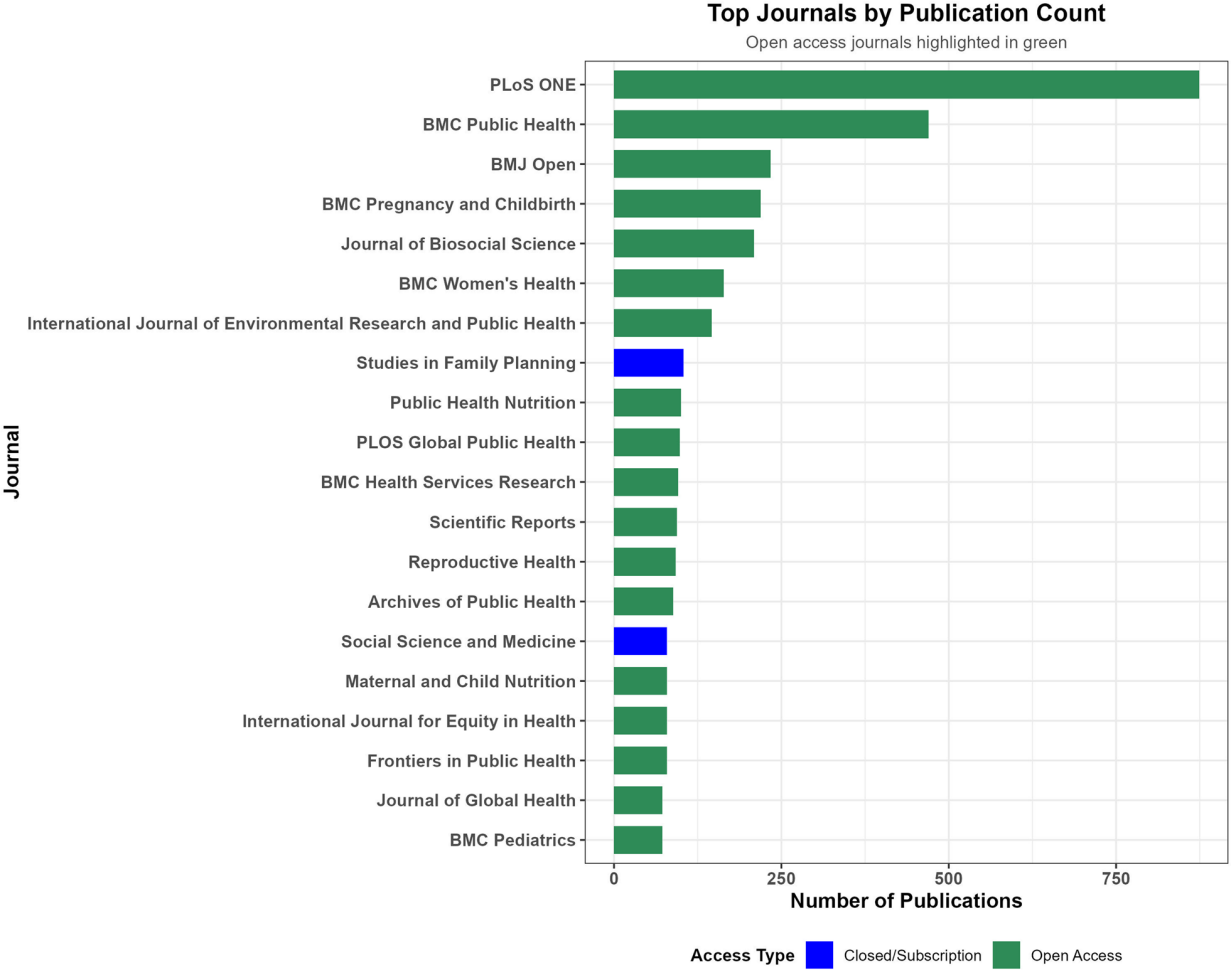
Top journals by publication count

Figure 5 presents the distribution of DHS-based articles across leading peer-reviewed journals. The results indicate that a number of open-access journals account for the majority of DHS research output. Journals such as *PLOS One*, *BMC Public Health* and *BMJ Open* emerge as the most frequent publishers, reflecting the growing preference for open-access dissemination within public health research. The prominence of these journals also suggests that DHS-based studies are reaching broad, international audiences, facilitating wider uptake among researchers, practitioners and policy actors.

**Table 1:** Most frequently mentioned funding organizations

Funding organization	Country	Institutional type	Mentions	%
National Intitutes of Health (NIH)	USA	Governmental	887	10.0
US Agency for International Development	USA	Governmental	447	5.1
Bill and Melinda Gates Foundation	USA	Philanthropic	386	4.4
UK Research and Innovation	UK	Governmental	271	3.1
Wellcome Trust	UK	Philanthropic	177	2.0
National Natural Science Foundation of China	China	Governmental	101	1.1
World Health Organization	Multilateral	Multilateral	96	1.1
Measure DHS	USA	Governmental	92	1.0
ICF International	USA	Corporate	84	1.0
Demographic and Health Survey	USA	Governmental	68	0.8
UNICEF	Multilateral	Multilateral	68	0.8
National Science Foundation	USA	Governmental	67	0.8
European Commission	European Union	Governmental	64	0.7
Intensive Care Foundation	Australia	Philanthropic	61	0.7
Department for International Development, UK Government	UK	Governmental	59	0.7
World Bank Group	Multilateral	Multilateral	56	0.6
United Nations Population Fund	Multilateral	Multilateral	52	0.6
National Research Foundation	South Africa	Governmental	49	0.6
National Health and Medical Research Council	Australia	Governmental	42	0.5
South African Medical Research Council	South Africa	Governmental	38	0.4
Deutsche Forschungsgemeinschaft (The German Research Foundation)	Germany	Governmental	34	0.4
Styrelsen för Internationellt Utvecklingssamarbete, Sida (Swedish International Development Cooperation Agency)	Sweden	Governmental	34	0.4

Funding information for DHS-based publications was extracted from the funding acknowledgements sections of the included articles. Where available, all explicitly named funding organizations were recorded for each publication. As a result, individual publications could contribute more than one funding acknowledgment, and Table 1 therefore reflects the frequency of funder mentions rather than the number of uniquely funded publications. A substantial proportion of DHS-based publications reported at least one external funding source, with many acknowledging multiple funders, reflecting the collaborative and often multisponsor nature of population health research. Funding organizations were subsequently classified by country and institutional type (governmental agencies, philanthropic foundations and multilateral organizations) to allow assessment of geographic patterns in research support.

The distribution of funders indicates that the majority of financial support originates from high-income country (HIC)-based institutions, including government agencies, research councils and philanthropic foundations, alongside a smaller but notable contribution from multilateral organizations. This funding landscape is particularly relevant when interpreted alongside authorship patterns, as DHS-based research frequently involves collaborations between HIC-based funders and researchers and institutions based in low- and middle-income countries (LMICs). These patterns emphasize the importance of examining how funding structures may shape research production, leadership and downstream academic and policy influence in subsequent analyses.

### Citation and influence analysis

#### Global publications versus citations

**Fig. 6:**
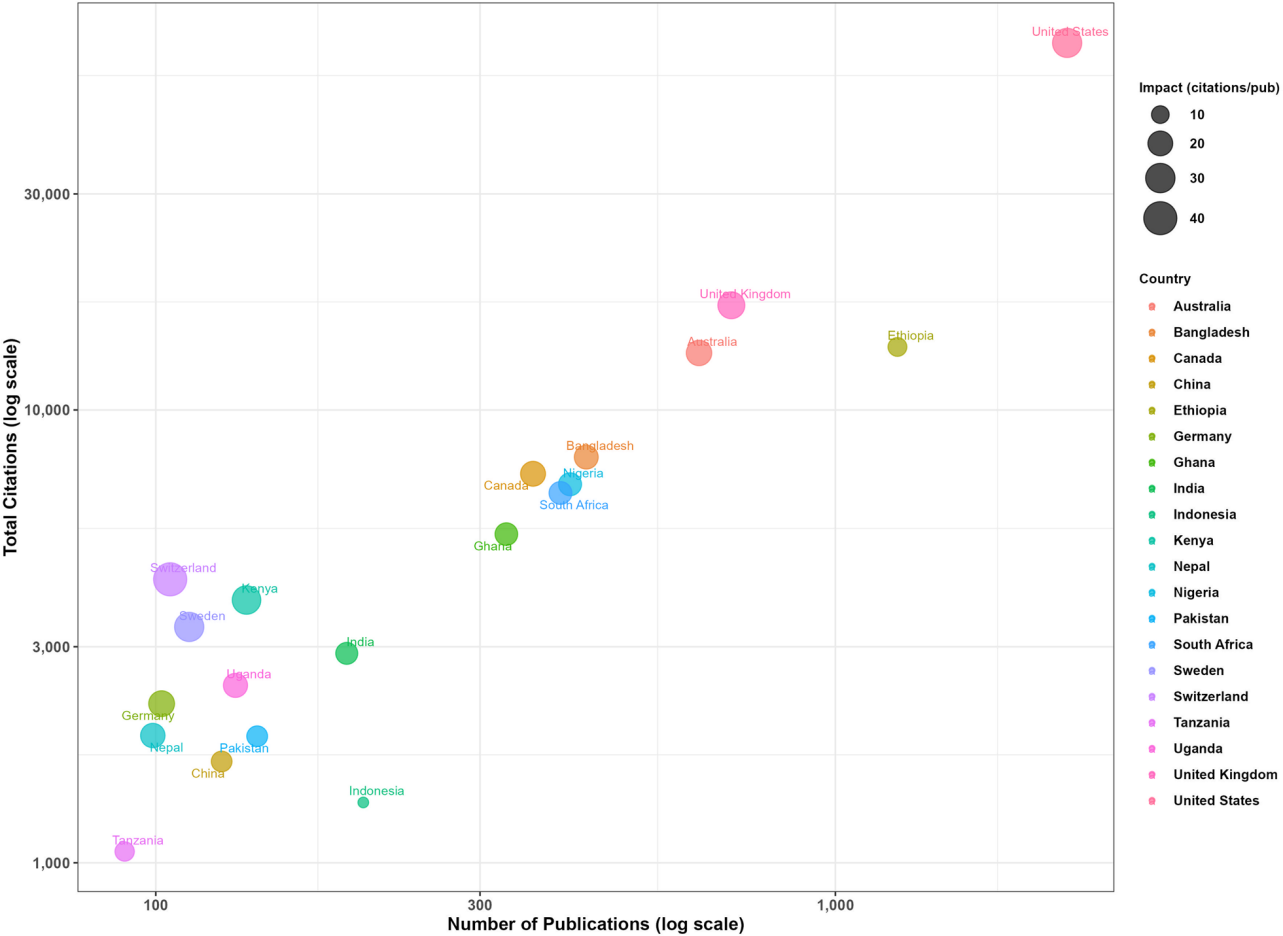
Global disparities in DHS research influence among top contributing countries

Figure 6 presents the relationship between publication output and total citations for the top 20 contributing countries, with bubble size indicating research impact (mean citations per paper). The results reveal clear global disparities in global scientific influence. The USA dominates, producing the largest volume of DHS-based publications and recording the highest citation counts and research impact. The UK and Australia also perform strongly, reflecting well-established scientific capacity. Among LMICs, Ethiopia, Bangladesh, Nigeria and South Africa stand out as leading contributors, with Ethiopia achieving a comparatively high citation rate per publication-evidence of strong visibility despite smaller output. A second tier of contributors, including Germany, Switzerland, Sweden and Canada, shows moderate yet consistent productivity. Finally, countries such as Tanzania, Nepal, Indonesia and Pakistan appear at the lower end of both publications and citations, indicating emerging capacity or more limited integration into global scholarly networks. Overall, the figure highlights persistent North–South imbalances alongside the rise of new research hubs in Africa and Asia. The positive association between publication volume and citations suggests cumulative advantage, though some LMICs demonstrate that targeted, high-quality research can still achieve significant influence.

#### Publications versus citations

**Fig. 7:**
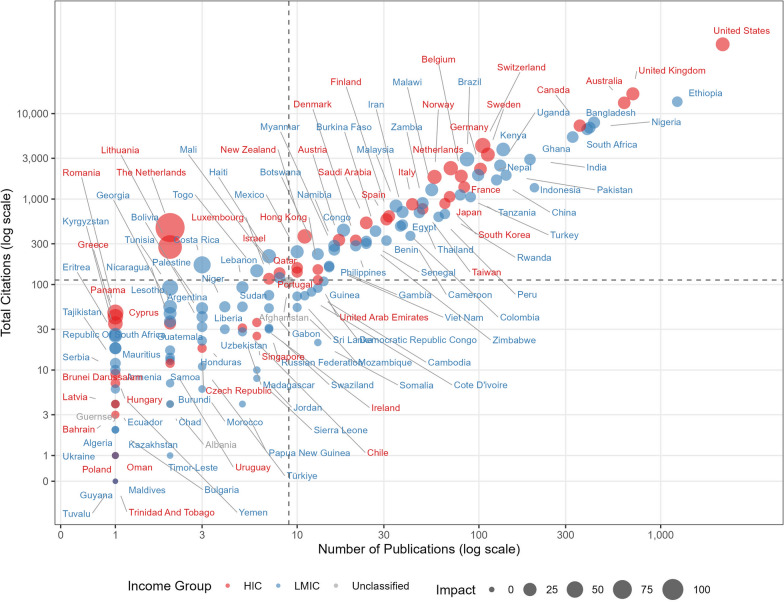
Scientific impact between high-income and low-and-middle-income countries

Figure 7 compares research productivity and scholarly influence between HICs (shown in red) and LMICs (shown in blue), on the basis of the logarithmic number of publications (*x*-axis) and total citations (*y*-axis). Bubble size represents average citation impact. A strong positive correlation appears between publication volume and citation counts, indicating that more publications are associated with higher citations. Nonetheless, considerable disparity persists between and within HICs and LMICs. Within HICs the USA, the UK, Australia and Canada emerge as leading contributors, producing relatively large publication outputs alongside large citations. Within LMICs, Ethiopia and Bangladesh are leading with high publication volume and citation impact. Positioned predominantly in the upper-right quadrant, these countries function as regional research hubs. Overall, the pattern reveals a widening research impact disparity between HICs and LMICs: most HICs are dominating, while the majority of LMICs remain underrepresented in global publication volume and citation impact.

**Fig. 8:**
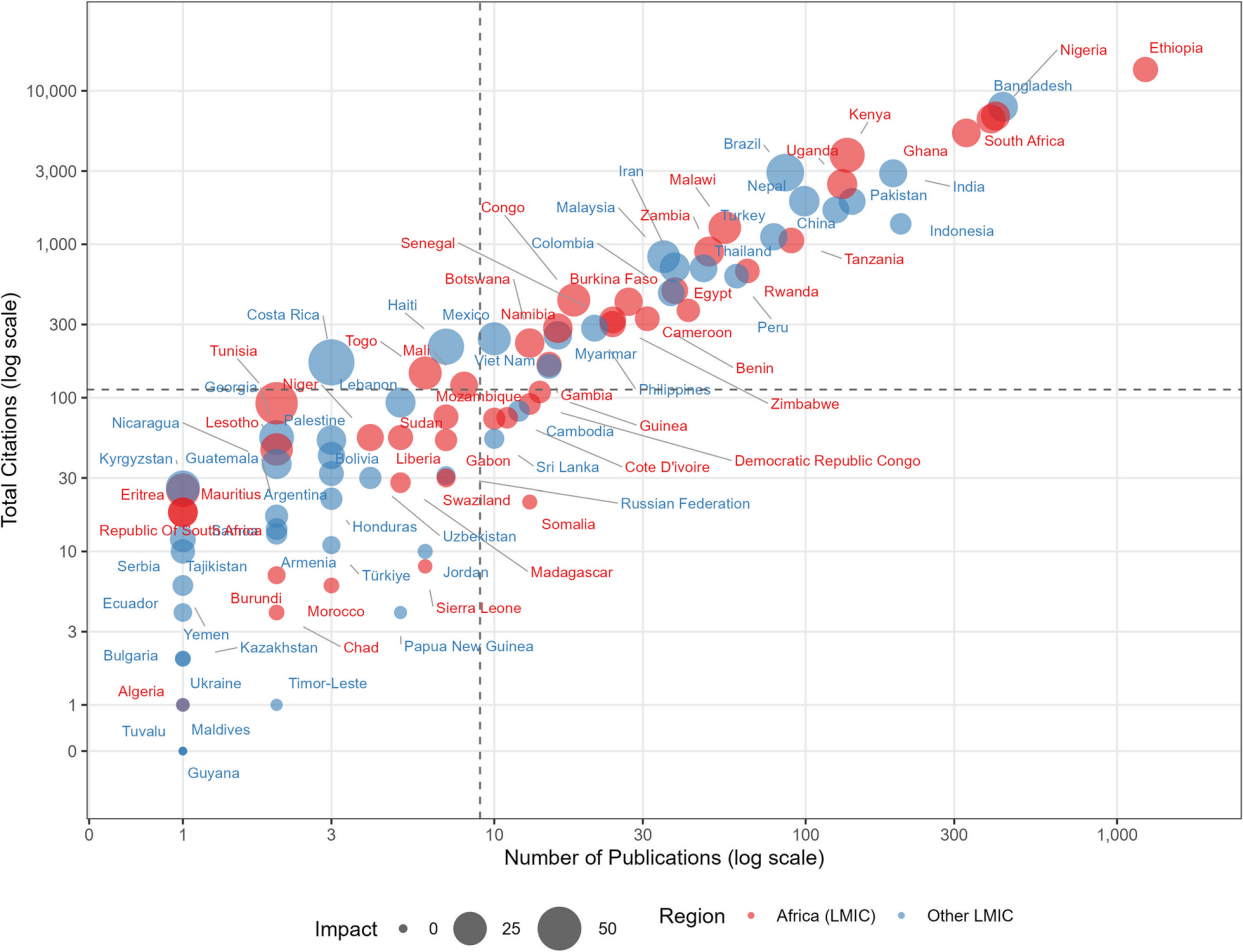
Research productivity and citation accrual among LMIC subgroups. Within the LMIC category, African-led studies show comparable publication growth to other LMIC regions but demonstrate lower cumulative citation counts

Figure 8 compares research output (publications) and influence (citations) among African and non-African LMICs. Overall, more publications are associated with more citations, but there is wide variation between countries. A small group of African LMICs – especially Ethiopia, Nigeria, South Africa, Ghana, Kenya and Uganda – stand out with high productivity and strong citation impact, indicating their role as regional research hubs. Most other African LMICs cluster at low publication and citation levels, while other LMICs such as India, Bangladesh, Brazil and Indonesia show notably higher publication and citation impact. Overall, the distribution highlights a widening intra-LMIC research gap: while a few African countries are emerging as influential research centers, the majority remain underrepresented in global scholarship. The size and position of bubbles further suggest that research impact in African LMICs is concentrated among a small subset of countries, emphasizing the need for targeted capacity-building and equitable research investment across the continent.

### Policy impact

**Fig. 9:**
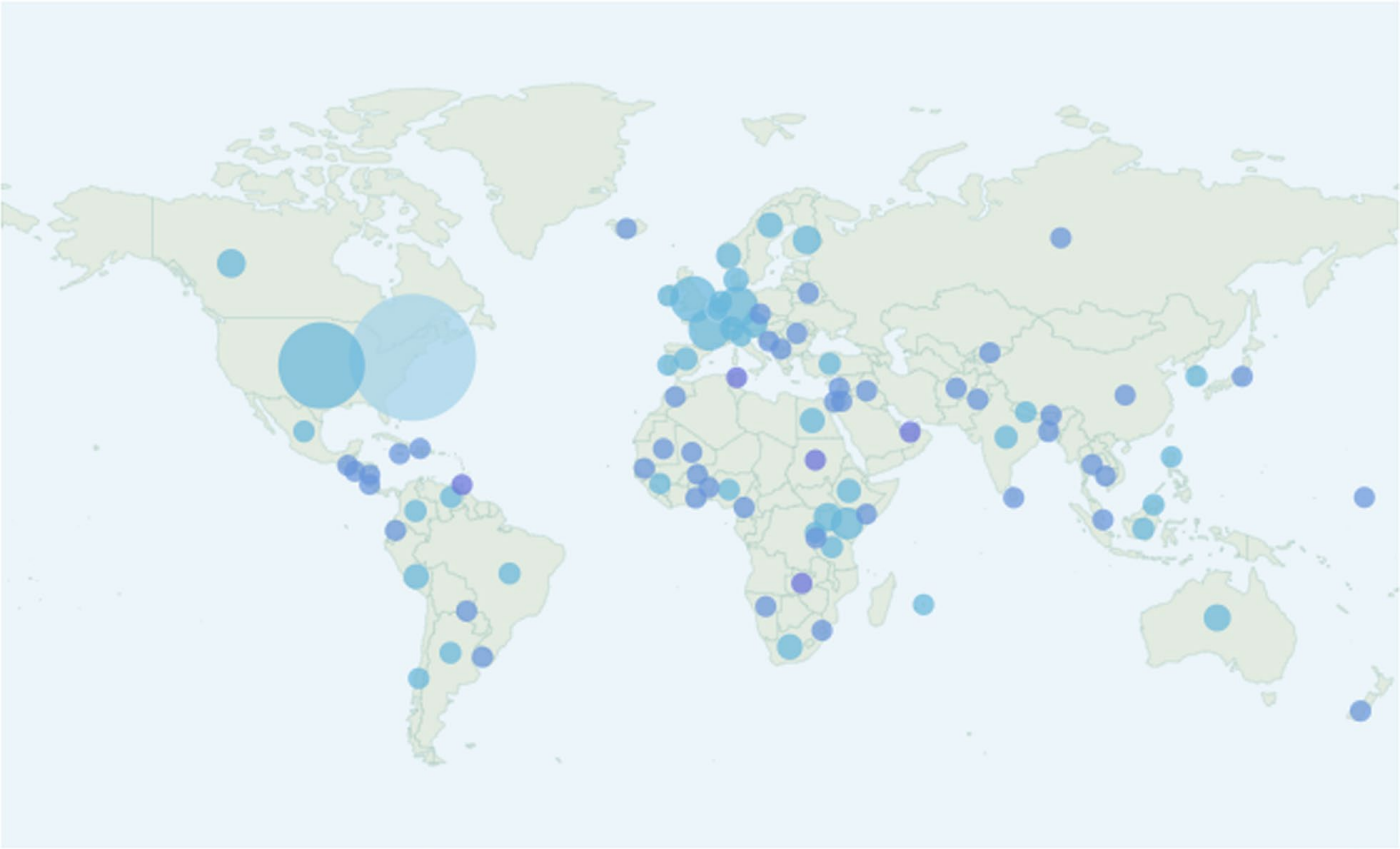
Citing policy countries of the 3073 articles. The cited countries are 94 with the articles cited in 553 sources as analysed by Overton

In Fig. 9, of those 10 267 DOIs (articles), Overton finds 3073 of the articles cited in policy (that is, about 30% of them), with intergovernmental organization (IGO) in USA/Canada accounting for 1981, USA accounting for 1228, UK 476, France 394, Germany 378, Kenya 210, Uganda 146, Canada 143, Finland 140, Australia 113, South Africa 90, Austria 85, Peru 81, Sweden 81, Egypt 78, Norway 77, Denmark 73, European Union (EU) 61, Ethiopia 61, Switzerland 59, Spain 43, India 40, Netherlands and Colombia accounting for 26 each, Indonesia 25, Tanzania and Turkey accounting for 23 each, Argentina and Brazil 20 each, Venezuela 18, Malaysia and South Korea 16 each, Nigeria, Nepal and Italy 13 each, Mexico 12, Belgium, Guinea, Ireland and Philippines 10 each, Rwanda 9, Portugal 8, Chile and Mauritius 7 each, Ghana and New Zealand 6 each, Pakistan and Jamaica 5 each, Singapore and Bangladesh 4 each, Bhutan, Cameroon, Czech Republic, Namibia, Russia and Sri Lanka 3 each, Japan, Uruguay, Thailand, Lebanon, Romania, Iraq, Burundi, Eswatini, Afghanistan and Cambodia 2 each and United Arab Emirates, Tunisia, Trinidad and Tobago, Sudan, Kyrgyzstan, Somalia, Senegal, Paraguay, Nicaragua, Morocco, Mauritania, Marshall Islands, Mali, China, Kosovo, Jordan, Israel, Iceland, Haiti, Guatemala, El Salvador, Ecuador, Costa Rica, Burkina Faso, Bosnia and Herzegovina, Benin, Belarus and Zambia 1 each. Note that 73.5% of these articles are cited more than once in policy.

**Table 2:** Citing policy source sector

Public sector	Private sector	Third sector
2469	25	1743

Analysis of the policy sources citing DHS-based research shows that citations are heavily concentrated within both the public and third sectors. As shown in Table 2, the public sector accounts for the largest share of citations (*n* = 2469), indicating that government ministries, public agencies and state-affiliated bodies remain the dominant users of DHS evidence. A substantial number of citations also originate from the third sector (*n* = 1743), which includes nongovernmental and civil society organizations. In contrast, only a small number of citations come from the private sector (*n* = 25), suggesting relatively limited uptake of DHS research in commercial or industry-driven policy publications.

**Table 3:** Citing organization types

Organization type	Articles
IGO	1988
Think tank	1642
Government	1021
Nongovernmental organization	478
Legistative body	49
University	2
Judicial body	1

Table 3 further disaggregates these sources by organizational type, showing that intergovernmental organizations (IGOs) are the most frequent citers (*n* = 1988). Think tanks are the second most common source (*n* = 1642), reflecting the use of DHS evidence in applied policy analysis and advocacy work. National government bodies also contribute significantly (*n* = 1021), suggesting that DHS research plays a notable role in informing state-level decision-making. Citation frequencies are notably lower for other organization types. Non-governmental organizations (*n* = 478) account for a smaller but still meaningful portion of usage, while legislative bodies (*n* = 49) and academic universities (*n* = 2) make only marginal contributions. Judicial bodies appear least represented, with just one citation recorded. These patterns show that DHS-based research utilization in policy arena is uneven across organizational domains, with the strongest uptake being among intergovernmental actors, think tanks and government agencies.

Supplementary Tables S2–S6 highlight the broad policy influence, thematic diversity and institutional engagement associated with DHS-based research. As presented in Supplementary Table S2, global policy institutions were the most prominent sources citing DHS-related publications, with the World Bank (828 articles), World Health Organization (807 articles) and United Nations (498 articles) accounting for the largest shares of citations, indicating strong uptake of DHS evidence in international policy processes. Supplementary Table S3 further demonstrates that the policy discourse surrounding DHS research is largely centered on health and development themes, particularly health (2784 articles), public health (1921), poverty (1917) and health care (1808), alongside topics such as maternal and child health, family planning and malnutrition. Consistent with this thematic focus, Supplementary Table S4 shows that the majority of citing policies fall within the domains of health, science and technology and education, reflecting the interdisciplinary application of DHS data across policy sectors. The disciplinary spread of the underlying research, presented in Supplementary Table S5, indicates that DHS-related publications are predominantly situated within public health, demography and health policy, although contributions also extend to development studies, economics, nutrition and infectious disease research. Finally, Supplementary Table S6 reveals a geographically diverse network of contributing institutions, with leading affiliations including Johns Hopkins University, London School of Hygiene and Tropical Medicine and Harvard University, alongside several institutions in low- and middle-income countries such as African Population and Health Research Center and Makerere University. These findings emphasize the extensive global reach of DHS-based research and its central role in informing health and development policy discussions.

**Fig. 10:**
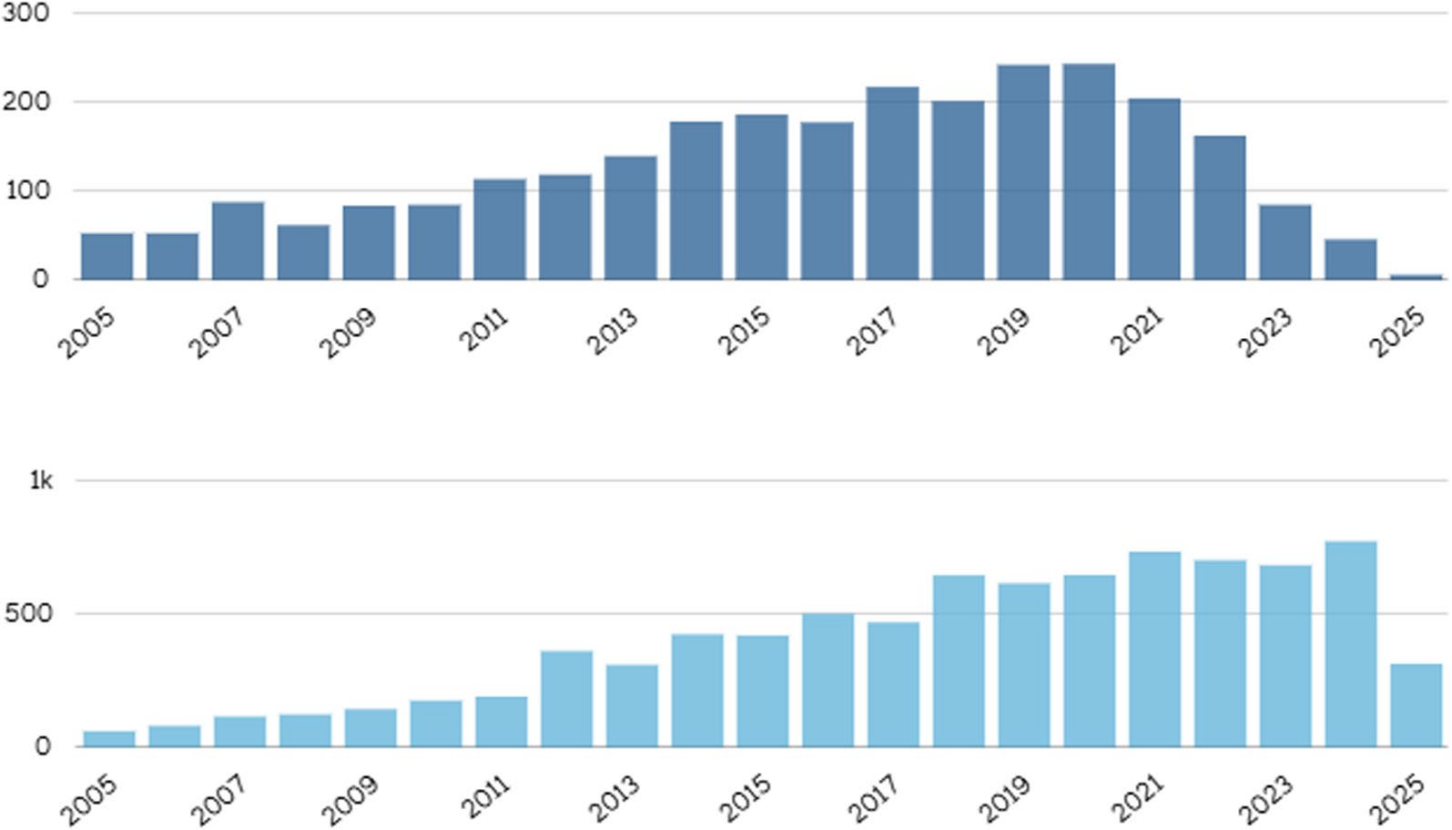
Scholarly article publication years (top panel) and citing policy publication years (bottom panel)

Figure 10 compares scholarly DHS article publications and DHS article citations in policy documents from 2005 to 2025. The DHS-based publications increased steadily from 2005 to 2025 (top panel). After modest article output in the mid-2000s, DHS publications began rising around 2010 and accelerated after 2015, peaking in the early 2020s, indicating a sustained and expanding academic utilization of DHS data. Similarly, DHS articles citations in policy publications also rose over time, although with a lower volume compared with academic articles. Early citations in policy documents (2005–2010) were limited, but a steady increase is visible from 2012, with noticeable peaks in the early 2020s, indicating expanding influence of DHS-based research within policy arenas. Although policy citations lag academic publications in absolute numbers, the upward growth suggests increasing translation of research evidence into policy processes.

## Discussion

Over the past four decades, the volume of DHS-based research has grown substantially. However, despite this growth, questions remain about how effectively such research translates into measurable academic and policy influence. This study was designed to examine these patterns at scale, recognizing that understanding how research circulates within scholarly and policy arenas is essential for strengthening evidence-informed decision-making and societal relevance [8]. The observed upward trends in both academic citations and policy document references indicate that publications drawing on DHS data constitute an increasingly visible component of the global public health evidence base. Importantly, the publications analysed in this study include research that used DHS data as either a primary data source or a significant secondary data source, often alongside other datasets or methodological inputs. Consequently, our findings do not imply that DHS-based research is uniquely preferred by policy-makers over other forms of evidence. Rather, they demonstrate that DHS-derived evidence features prominently within a broader ecosystem of research that informs academic discourse and policy-relevant analysis.

The growth in citations is likely influenced by multiple contributing factors, including expanded survey coverage across countries and time, improved access to DHS microdata, enhanced documentation and harmonization of indicators and increasing methodological sophistication among researchers analysing DHS data [30]. These developments have strengthened the integration of DHS-based findings into comparative analyses, trend assessments and equity-focused research that are frequently drawn upon in both scholarly and policy-oriented contexts [30]. The citation peaks arguably correspond with major global health milestones. For instance, DHS research accelerated during the MDG era (2000–2015), when reproductive, maternal and child health and HIV/AIDs were priority areas [25]. The surge after 2015 and peaks in early 2020s coincides with the Sustainable Development Goals (SDGs), where DHS indicators are embedded in monitoring frameworks for health, nutrition, gender inequality and population dynamics [31]. As governments and development partners intensify efforts to measure progress and inequalities in coverage indicators, DHS serves as a primary data source [32]. These policy milestones have therefore not only increased the production of DHS-based research but also amplified its citation impact across academic and policy spheres [30].

The three journals with the highest number of DHS-based publications, *PLOS One*, *BMC Public Health* and *BMJ Open*, are all fully open-access journals and were among the most frequent publication venues identified in our bibliometric analysis. These journals are comparatively more accessible to researchers from low- and middle-income countries owing to full, partial or conditional article processing charge (APC) waiver policies [33]. For example, *BMJ Open* offers full APC waivers for authors from all LMICs, while BMC Public Health provides full waivers for low-income countries and partial waivers for lower–middle-income countries. *PLOS One* offers conditional publication fee assistance, although the application-based nature of this support and the absence of guaranteed approval may still present barriers for several LMIC researchers [34]. Our findings suggest that journals with broader disciplinary scope and more flexible APC policies serve as key outlets for DHS-based research, likely facilitating higher publication volumes and subsequent citation accrual. This pattern aligns with existing evidence showing that open-access publishing increases visibility and citation impact, particularly for research focused on low- and middle-income settings, while author-facing charges can simultaneously introduce new inequities in who is able to publish [35].

In contrast, high-impact general medical journals such as *The Lancet* and *JAMA*, which typically do not offer routine APC waivers for LMIC authors, did not feature among the leading publication venues for DHS-based primary research in our dataset. This observation is consistent with prior analyses documenting the underrepresentation of LMIC-led research in prestigious journals and identifying article processing charges, limited publication fee support and reliance on grant-covered APCs as important structural barriers. While several DHS-based studies are published in journals without guaranteed waivers, often through external funding or collaborative arrangements, this model advantages well-resourced institutions and disadvantages researchers without access to APC funding, reinforcing existing global inequities in scholarly publishing [33, 35–37].

Citation growth for primary DHS publications has expanded across many journals, likely reflecting an evolving and increasingly diversified funding landscape. In the early years, DHS-based research relied predominantly on a small number of major funders such as the US Agency for International Development (USAID), United Nations (UN) agencies and the World Bank. Over time, additional major funders including the Bill and Melinda Gates Foundation, the Wellcome Trust and the Eunice Kennedy Shriver National Institute of Child Health and Human Development became increasingly involved, indicating a shift in the composition rather than a simple increase in the overall volume of investment. Their engagement was often motivated by alignment between DHS survey modules and specific thematic priorities (for example, malaria and infectious diseases for the Wellcome Trust and women’s and reproductive health for the Gates Foundation), as well as by broader global health initiatives such as the expansion of WHO HIV/AIDS programmes, the establishment of the Global Fund and increased attention to noncommunicable diseases [38]. These changes in funding priorities are reflected in the widening scope of DHS surveys, which initially focused on family planning and contraception but later incorporated modules on domestic violence, HIV/AIDS and other emerging health concerns [30].

While our analysis does not quantify funding volumes over time, patterns observed in funding acknowledgements suggest that investment in DHS-based research has become more pluralistic and distributed across a wider set of funding actors, supporting secondary data analysis, country-initiated projects, methodological innovation and publication costs. This diversification of support likely contributed to the steady rise in DHS-based publications, citations and policy-relevant outputs observed in this study, rather than reflecting a uniform increase in financial investment alone [39]. In the context of USAID’s recent withdrawal of funding in 2025, this broader donor base becomes strategically important. The engagement of both traditional global funders and newer philanthropic and research-focused partners offers pathways for sustaining DHS surveys and maintaining open-access data availability, as illustrated by recent interim support from the Gates Foundation and other partners [39].

Approximately one third of citations to primary DHS-based articles are found within policy-relevant documents, indicating that DHS-derived research evidence is being taken up across intergovernmental organizations, think tanks, government agencies and nongovernmental organizations. These citing documents are predominantly produced by actors in the public and third sectors, where empirical population-based evidence is commonly used to inform agenda setting, policy analysis, programme design and accountability frameworks, rather than constituting final policy decisions [40]. By contrast, relatively few policy documents originating from private-sector organizations cite DHS-based scientific publications. This pattern likely reflects differences in how evidence is generated and used across sectors, as well as the heterogeneity of private-sector actors involved in health, many of whom rely on proprietary data, market-driven analyses or operational evidence rather than publicly available survey data. This more limited use of DHS-based research is notable given that the private sector is widely recognized as an important partner in advancing universal health coverage alongside public institutions [41, 42], highlighting a potential gap in the integration of population-level survey evidence across sectors. Strengthening regulatory frameworks, public–private research partnerships and shared financing mechanisms may enhance opportunities for incorporating DHS-derived evidence into private-sector-engaged policy processes [43].

These findings further underscore the broad policy relevance and thematic range of DHS-based research. The high frequency of citations within policy documents produced by global institutions such as the World Bank, World Health Organization and United Nations suggests that research using DHS data is regularly drawn upon to support international health and development policy analysis and reporting. The prominence of themes related to health, poverty and maternal and child health is consistent with longstanding global development priorities, while the presence of DHS-based evidence across policy domains spanning health, science and education illustrates the versatility of DHS data for addressing complex, cross-sectoral development questions. Collectively, these patterns indicate that the DHS functions as a foundational data resource for researchers whose analytical outputs are subsequently cited within policy-relevant documents. In this sense, DHS-based research contributes to both scientific advancement and policy discourse by supplying standardized, comparable evidence that can be mobilized by a range of actors within global and national policy environments, reinforcing the programme’s role as a global public good.

However, geographical inequities were observed in the policy uptake of DHS-based research, with policy document citations concentrated predominantly in the USA and Europe, primarily through intergovernmental organizations, think tanks and internal government agencies. Although DHS-based research is cited in policy-relevant documents from several countries in Africa particularly in East Africa, the overall volume of such citations remains comparatively low. This pattern does not necessarily indicate slower translation of evidence in African or other low- and middle-income countries (LMICs) but may instead reflect structural and contextual differences in policy documentation practices, research–policy interfaces and the coverage of policy documents captured by bibliometric databases. More generally, studies of research translation suggest that the movement of evidence into policy and practice can extend over long time horizons, with average lags of more than a decade observed across a range of settings and disciplines, predominantly based on evidence from high-income countries [44]. The relatively limited representation of LMIC-produced policy documents in citation-based analyses may therefore be influenced by a combination of factors, including differential research capacity, fewer formal policy outputs, variations in citation practices and limitations in the indexing of policy literature from LMIC contexts. Given the increasing volume of DHS-based research over time, these findings underscore the importance of strengthening capacity for policy-relevant research synthesis, communication and engagement with policy-makers within LMIC institutions, to enhance the visibility and potential uptake of locally generated evidence [45].

### Strength and limitations

This study has notable strengths, including a contemporary and extensive search strategy that updates previous reviews in the field. It also addresses a critical gap by focusing on the policy impact and citation influence of DHS outputs, an aspect neglected in past analyses. The use of multiple databases strengthened the comprehensiveness of the scientific literature identified. Despite these strengths, the study is subject to several cogent limitations. First, although the DHS programme has collected, analysed and disseminated high-quality, nationally representative data on population, health, HIV and nutrition through more than 400 surveys across more than 90 countries, the approach applied to identify relevant publications may not fully capture evidence related to all these key thematic areas. This is because several studies draw on DHS data but reference it only through specific thematic indicators, without explicitly mentioning the DHS as the data source. As a result, some research utilizing DHS data, especially studies focusing on thematic variables, may have been missed. This may have resulted in an underestimation of the true volume of DHS-based research and its citation and policy influence. The second limitation relates to the uneven availability of recent DHS data across countries. Not all countries have up-to-date data, with several having gaps since their last survey. For example, Mexico’s most recent DHS dates to 1987, while Colombia’s last survey was conducted in 2015. Similar gaps exist in several low- and middle-income countries in Latin America and the Caribbean. Therefore, countries without recent DHS data are less likely to generate recent publications and consequent citations and policy influence. Third, the assessment of policy impact was constrained by its dependence on a single data source, Overton, which may have led to an undercount of relevant policy citations. Furthermore, Overton database may not provide a fully representative coverage of policy documents from low- and middle-income countries, meaning that policy citations from these settings are likely undercounted, which may partially explain the comparatively lower volume of DHS-based policy citations observed for LMICs. Moreover, we did not conduct a subject-area analysis because initial natural language processing yielded a vast and redundant set of themes that were not viable for systematic categorization.

## Conclusions

This analysis confirms that DHS outputs are playing an increasingly critical role in shaping both scholarly and policy-oriented publications. While DHS-based research shows substantial academic reach and citation growth, its uptake within policy-relevant documents is uneven across geographic contexts more particularly in LMIC countries. This disparity underscores the need for initiatives that bolster knowledge translation capabilities within LMIC institutions, particularly those aimed at cultivating private-sector partnerships. Concurrently, the broader scientific community must address structural barriers by advocating for equitable publishing models that do not rely on author fees. Ultimately, future studies are needed to delve deeper into thematic policy impacts and to quantify the real-world influence of this research within individual countries. Furthermore, there is need for future research to explore how characteristics of academic publications influence the translation of evidence into policy. In particular, examining cross-tabulations between article attributes such as research topics, country focus, institutional affiliations, funding sources and collaboration networks and their uptake in policy documents could provide deeper insights into the mechanisms through which research informs policy processes. Such analyses would help identify institutional configurations, funding patterns and collaborative arrangements that are associated with greater policy visibility and impact of DHS-based research. Understanding these dynamics would be valuable for researchers, funders and policy actors seeking to strengthen the policy relevance of population health research. As bibliometric and policy tracking databases continue to improve in coverage and metadata integration, future studies will be better positioned to conduct these analyses and generate actionable evidence on how research investments and collaborations can enhance the policy uptake of DHS-derived evidence.

## Additional file

Supplementary file 1.

## Data Availability

No datasets were generated or analysed during the current study.
