# Supplementary material for: Citation and policy influence of research using demographic and health survey data: a bibliometric analysis
Source: Health Res Policy Syst. 2026 May 14;24:61. doi: 10.1186/s12961-026-01487-0 (PMC13371176; doi:10.1186/s12961-026-01487-0)
Supplement: Supplementary file 1 [file 12961_2026_1487_MOESM1_ESM.docx]

| **Supplementary Table S1. Detailed search strategy in databases.** | |
| --- | --- |
| **Database** | **Search strategy** |
| Scopus  (12279) | TITLE-ABS-KEY (“Demographic and Health Surveys” OR “Demographic Health Program” OR “Demographic Health Data” OR “Demographic Health Study” OR (Demograph* AND “Health Survey”)) |
| PubMed  (13108) | (((“Demographic and Health Surveys” [All Fields]) OR (“Demographic Health Program” [All Fields]) OR (“Demographic Health Data” [All Fields]) OR (“Demographic Health Study” [All Fields])) OR ((Demograph*[All Fields]) AND (“Health Survey” [All Fields]))) |
| Web of Science  (14222) | S= (“Demographic and Health Surveys” OR “Demographic Health Program” OR “Demographic Health Data” OR “Demographic Health Study” OR (Demograph* AND “Health Survey”)) |
| Dimension  (500) | Abstract and Title: (“Demographic and Health Surveys” OR “Demographic Health Program” OR “Demographic Health Data” OR “Demographic Health Study” OR (Demograph* AND “Health Survey”)) |
| Cinahl  (11877) | (MH “Demographic and Health Surveys”) OR (TX “Demographic Health Program”) OR (TX “Demographic Health Data”) OR (TX “Demographic Health Study”) OR ((TX Demograph*) AND (TX “Health Survey”)) |
| Wiley  (1658) | (“Demographic and Health Surveys” OR “Demographic Health Program” OR “Demographic Health Data” OR “Demographic Health Study” OR (Demograph* AND “Health Survey”)) |
|  | |

**Supplementary Table S2: Sources of citing policy**

| **Country/Body** | **Sources** | **Articles** |
| --- | --- | --- |
| Global | [World Bank](https://app.overton.io/articles.php?identifiers=set%3A392%3A0f5c3195569f8372a21a47ce977169e8&format=report&sort=date&format=&cited_by_policy_sources=worldbank&get_ids=doi&passthru_source=worldbank)  Public Sector  IGO  International Body, Financial Institution or Bank, Development Bank, Policy Centre | 828 |
| Global | [World Health Organization](https://app.overton.io/articles.php?identifiers=set%3A392%3A0f5c3195569f8372a21a47ce977169e8&format=report&sort=date&format=&cited_by_policy_sources=who&get_ids=doi&passthru_source=who)  Public Sector  IGO  International Body, Top-Level Authority, Healthcare Service, Body or Agency, Research Centre, Policy Centre | 807 |
| Global | [United Nations](https://app.overton.io/articles.php?identifiers=set%3A392%3A0f5c3195569f8372a21a47ce977169e8&format=report&sort=date&format=&cited_by_policy_sources=unitednations&get_ids=doi&passthru_source=unitednations)  Public Sector  IGO  International Body, Top-Level Authority, IGO Department or Agency, Mixed Roles, Policy Centre | 498 |
| United States of America | [Government Publishing Office (GPO)](https://app.overton.io/articles.php?identifiers=set%3A392%3A0f5c3195569f8372a21a47ce977169e8&format=report&sort=date&format=&cited_by_policy_sources=gpogov&get_ids=doi&passthru_source=gpogov)  Public Sector  Government  National Body, Mixed Roles, Public Service | 399 |
| France | [CGIAR](https://app.overton.io/articles.php?identifiers=set%3A392%3A0f5c3195569f8372a21a47ce977169e8&format=report&sort=date&format=&cited_by_policy_sources=cgiar&get_ids=doi&passthru_source=cgiar)  Third Sector  Think Tank  Research Centre, Initiative, Programme or Project, Policy Centre, Professional Network, Association, Union or Cooperative | 395 |
| United States of America | [Population Council](https://app.overton.io/articles.php?identifiers=set%3A392%3A0f5c3195569f8372a21a47ce977169e8&format=report&sort=date&format=&cited_by_policy_sources=populationcouncilusa&get_ids=doi&passthru_source=populationcouncilusa)  Third Sector  NGO  Research Centre | 324 |
| Germany | [IZA Institute of Labor Economics](https://app.overton.io/articles.php?identifiers=set%3A392%3A0f5c3195569f8372a21a47ce977169e8&format=report&sort=date&format=&cited_by_policy_sources=izade&get_ids=doi&passthru_source=izade)  Third Sector  Think Tank  Research Centre, Policy Centre | 290 |
| United States of America | [IFPRI](https://app.overton.io/articles.php?identifiers=set%3A392%3A0f5c3195569f8372a21a47ce977169e8&format=report&sort=date&format=&cited_by_policy_sources=ifpri&get_ids=doi&passthru_source=ifpri)  Third Sector  Think Tank  Research Centre, Policy Centre | 237 |
| United Kingdon | [Institute of Development Studies](https://app.overton.io/articles.php?identifiers=set%3A392%3A0f5c3195569f8372a21a47ce977169e8&format=report&sort=date&format=&cited_by_policy_sources=instituteofdevelopmentstudies&get_ids=doi&passthru_source=instituteofdevelopmentstudies)  Third Sector  Think Tank  Research Centre, Policy Centre | 225 |
| Global | [UNICEF](https://app.overton.io/articles.php?identifiers=set%3A392%3A0f5c3195569f8372a21a47ce977169e8&format=report&sort=date&format=&cited_by_policy_sources=unicef)  Public Sector  IGO  International Body, IGO Department or Agency, Initiative, Programme or Project, Food and Drug Safety, Healthcare Service, Body or Agency | 188 |
| Global | [Food and Agriculture Organization of the United Nations](https://app.overton.io/articles.php?identifiers=set%3A392%3A0f5c3195569f8372a21a47ce977169e8&format=report&sort=date&format=&cited_by_policy_sources=fao)  Public Sector  IGO  International Body, IGO Department or Agency, Food and Drug Safety, Healthcare Service, Body or Agency | 159 |
| Finland | [United Nations University World Institute for Development Economics Research](https://app.overton.io/articles.php?identifiers=set%3A392%3A0f5c3195569f8372a21a47ce977169e8&format=report&sort=date&format=&cited_by_policy_sources=unuwider)  Public Sector  Think Tank  Research Centre, Policy Centre | 141 |
| Kenya | [African Economic Research Consortium](https://app.overton.io/articles.php?identifiers=set%3A392%3A0f5c3195569f8372a21a47ce977169e8&format=report&sort=date&format=&cited_by_policy_sources=aerckenya)  Third Sector  Think Tank  Research Centre, Policy Centre | 140 |
| United States of America | [NBER](https://app.overton.io/articles.php?identifiers=set%3A392%3A0f5c3195569f8372a21a47ce977169e8&format=report&sort=date&format=&cited_by_policy_sources=nber)  Third Sector  Think Tank  Research Centre | 136 |
| Uganda | [Government of Uganda](https://app.overton.io/articles.php?identifiers=set%3A392%3A0f5c3195569f8372a21a47ce977169e8&format=report&sort=date&format=&cited_by_policy_sources=governmentofuganda)  Public Sector  Government  National Body, Public Service, Mixed Roles | 131 |
| Global | [United Nations Population Fund](https://app.overton.io/articles.php?identifiers=set%3A392%3A0f5c3195569f8372a21a47ce977169e8&format=report&sort=date&format=&cited_by_policy_sources=unpopulation)  Public Sector  IGO  International Body, IGO Department or Agency, Healthcare Service, Body or Agency, Policy Centre, Initiative, Programme or Project | 128 |
| United Kingdom | [Overseas Development Institute (ODI)](https://app.overton.io/articles.php?identifiers=set%3A392%3A0f5c3195569f8372a21a47ce977169e8&format=report&sort=date&format=&cited_by_policy_sources=odi)  Third Sector  Think Tank  Research Centre, Policy Centre | 127 |
| Canada | [International Development Research Centre](https://app.overton.io/articles.php?identifiers=set%3A392%3A0f5c3195569f8372a21a47ce977169e8&format=report&sort=date&format=&cited_by_policy_sources=idrc)  Public Sector  Think Tank  Research Centre, Policy Centre | 118 |
| United States of America | [Center for Global Development](https://app.overton.io/articles.php?identifiers=set%3A392%3A0f5c3195569f8372a21a47ce977169e8&format=report&sort=date&format=&cited_by_policy_sources=cgdev)  Third Sector  Think Tank  Research Centre, Policy Centre | 113 |
| Global | [UNESCO](https://app.overton.io/articles.php?identifiers=set%3A392%3A0f5c3195569f8372a21a47ce977169e8&format=report&sort=date&format=&cited_by_policy_sources=unesco)  Public Sector  IGO  International Body, IGO Department or Agency, Cultural Institution, Policy Centre | 113 |
| United States of America | [Guidelines in PubMed Central](https://app.overton.io/articles.php?identifiers=set%3A392%3A0f5c3195569f8372a21a47ce977169e8&format=report&sort=date&format=&cited_by_policy_sources=pubmedcentral)  Third Sector  Think Tank  National Body, Mixed Roles, Healthcare Service, Body or Agency, Archive, Public Data Body or Statistics | 108 |
| Global | [Inter-American Development Bank](https://app.overton.io/articles.php?identifiers=set%3A392%3A0f5c3195569f8372a21a47ce977169e8&format=report&sort=date&format=&cited_by_policy_sources=iadb)  Public Sector  IGO  International Body, Financial Institution or Bank, Development Bank, Policy Centr | 92 |
| Global | [Asian Development Bank](https://app.overton.io/articles.php?identifiers=set%3A392%3A0f5c3195569f8372a21a47ce977169e8&format=report&sort=date&format=&cited_by_policy_sources=asiandevelopmentbank)  Public Sector  IGO  International Body, Financial Institution or Bank, Development Bank | 89 |
| Global | [OECD](https://app.overton.io/articles.php?identifiers=set%3A392%3A0f5c3195569f8372a21a47ce977169e8&format=report&sort=date&format=&cited_by_policy_sources=oecddiscovery)  Public Sector  IGO  International Body, IGO Department or Agency, Monitoring or Regulatory Body, Policy Centre, Research Centre | 87 |
| Austria | [IIASA](https://app.overton.io/articles.php?identifiers=set%3A392%3A0f5c3195569f8372a21a47ce977169e8&format=report&sort=date&format=&cited_by_policy_sources=iiasaat)  Third Sector  Think Tank  Research Centre, Policy Centre | 84 |
| United States of America | [International Center for Research on Women](https://app.overton.io/articles.php?identifiers=set%3A392%3A0f5c3195569f8372a21a47ce977169e8&format=report&sort=date&format=&cited_by_policy_sources=icrw)  Third Sector  Think Tank  Research Centre, Policy Centre | 81 |
| Australia | Australian Policy Online  Third Sector  NGO  Public Data Body or Statistics, Archive, Policy Centre | 79 |
| Egypt | Economic Research Forum  Third Sector  Think Tank  Research Centre, Policy Centre | 78 |
| Ethiopia | [Government of Ethiopia](https://app.overton.io/articles.php?identifiers=set%3A392%3A0f5c3195569f8372a21a47ce977169e8&format=report&sort=date&format=&cited_by_policy_sources=governmentofethiopia)  Public Sector  Government  National Body, Mixed Roles, Public Service | 75 |

**Supplementary Table S3: Topics of citing policy**

| **Name** | **Articles** |
| --- | --- |
| [Health](https://app.overton.io/articles.php?identifiers=set%3A392%3A0f5c3195569f8372a21a47ce977169e8&format=report&sort=date&format=&citing_topics=Health) | 2,784 |
| [Public health](https://app.overton.io/articles.php?identifiers=set%3A392%3A0f5c3195569f8372a21a47ce977169e8&format=report&sort=date&format=&citing_topics=Public%20health) | 1,921 |
| [Poverty](https://app.overton.io/articles.php?identifiers=set%3A392%3A0f5c3195569f8372a21a47ce977169e8&format=report&sort=date&format=&citing_topics=Poverty) | 1,917 |
| [Health care](https://app.overton.io/articles.php?identifiers=set%3A392%3A0f5c3195569f8372a21a47ce977169e8&format=report&sort=date&format=&citing_topics=Health%20care) | 1,808 |
| [Health sciences](https://app.overton.io/articles.php?identifiers=set%3A392%3A0f5c3195569f8372a21a47ce977169e8&format=report&sort=date&format=&citing_topics=Health%20sciences) | 1,744 |
| [Education](https://app.overton.io/articles.php?identifiers=set%3A392%3A0f5c3195569f8372a21a47ce977169e8&format=report&sort=date&format=&citing_topics=Education) | 1,657 |
| [Research](https://app.overton.io/articles.php?identifiers=set%3A392%3A0f5c3195569f8372a21a47ce977169e8&format=report&sort=date&format=&citing_topics=Research) | 1,572 |
| [Human activities](https://app.overton.io/articles.php?identifiers=set%3A392%3A0f5c3195569f8372a21a47ce977169e8&format=report&sort=date&format=&citing_topics=Human%20activities) | 1,433 |
| [Child](https://app.overton.io/articles.php?identifiers=set%3A392%3A0f5c3195569f8372a21a47ce977169e8&format=report&sort=date&format=&citing_topics=Child) | 1,402 |
| [Branches of science](https://app.overton.io/articles.php?identifiers=set%3A392%3A0f5c3195569f8372a21a47ce977169e8&format=report&sort=date&format=&citing_topics=Branches%20of%20science) | 1,398 |
| [Pregnancy](https://app.overton.io/articles.php?identifiers=set%3A392%3A0f5c3195569f8372a21a47ce977169e8&format=report&sort=date&format=&citing_topics=Pregnancy) | 1,365 |
| [Risk](https://app.overton.io/articles.php?identifiers=set%3A392%3A0f5c3195569f8372a21a47ce977169e8&format=report&sort=date&format=&citing_topics=Risk) | 1,345 |
| [Medical specialties](https://app.overton.io/articles.php?identifiers=set%3A392%3A0f5c3195569f8372a21a47ce977169e8&format=report&sort=date&format=&citing_topics=Medical%20specialties) | 1,321 |
| [World Health Organization](https://app.overton.io/articles.php?identifiers=set%3A392%3A0f5c3195569f8372a21a47ce977169e8&format=report&sort=date&format=&citing_topics=World%20Health%20Organization) | 1,298 |
| [Economy](https://app.overton.io/articles.php?identifiers=set%3A392%3A0f5c3195569f8372a21a47ce977169e8&format=report&sort=date&format=&citing_topics=Economy) | 1,288 |
| [Sustainable Development Goals](https://app.overton.io/articles.php?identifiers=set%3A392%3A0f5c3195569f8372a21a47ce977169e8&format=report&sort=date&format=&citing_topics=Sustainable%20Development%20Goals) | 1,266 |
| [Demographic and Health Surveys](https://app.overton.io/articles.php?identifiers=set%3A392%3A0f5c3195569f8372a21a47ce977169e8&format=report&sort=date&format=&citing_topics=Demographic%20and%20Health%20Surveys) | 1,249 |
| [Sampling (statistics)](https://app.overton.io/articles.php?identifiers=set%3A392%3A0f5c3195569f8372a21a47ce977169e8&format=report&sort=date&format=&citing_topics=Sampling%20%28statistics%29) | 1,233 |
| [Violence](https://app.overton.io/articles.php?identifiers=set%3A392%3A0f5c3195569f8372a21a47ce977169e8&format=report&sort=date&format=&citing_topics=Violence) | 1,200 |
| [Malnutrition](https://app.overton.io/articles.php?identifiers=set%3A392%3A0f5c3195569f8372a21a47ce977169e8&format=report&sort=date&format=&citing_topics=Malnutrition) | 1,194 |
| Regression analysis | 1,194 |
| [Medicine](https://app.overton.io/articles.php?identifiers=set%3A392%3A0f5c3195569f8372a21a47ce977169e8&format=report&sort=date&format=&citing_topics=Medicine) | 1,186 |
| [Gender](https://app.overton.io/articles.php?identifiers=set%3A392%3A0f5c3195569f8372a21a47ce977169e8&format=report&sort=date&format=&citing_topics=Gender) | 1,184 |
| [Childbirth](https://app.overton.io/articles.php?identifiers=set%3A392%3A0f5c3195569f8372a21a47ce977169e8&format=report&sort=date&format=&citing_topics=Childbirth) | 1,177 |
| [Family planning](https://app.overton.io/articles.php?identifiers=set%3A392%3A0f5c3195569f8372a21a47ce977169e8&format=report&sort=date&format=&citing_topics=Family%20planning) | 1,146 |
| [Family](https://app.overton.io/articles.php?identifiers=set%3A392%3A0f5c3195569f8372a21a47ce977169e8&format=report&sort=date&format=&citing_topics=Family) | 1,141 |
| [Maternal death](https://app.overton.io/articles.php?identifiers=set%3A392%3A0f5c3195569f8372a21a47ce977169e8&format=report&sort=date&format=&citing_topics=Maternal%20death) | 1,119 |
| [Woman](https://app.overton.io/articles.php?identifiers=set%3A392%3A0f5c3195569f8372a21a47ce977169e8&format=report&sort=date&format=&citing_topics=Woman) | 1,109 |
| [Sustainability](https://app.overton.io/articles.php?identifiers=set%3A392%3A0f5c3195569f8372a21a47ce977169e8&format=report&sort=date&format=&citing_topics=Sustainability) | 1,103 |
| [Statistics](https://app.overton.io/articles.php?identifiers=set%3A392%3A0f5c3195569f8372a21a47ce977169e8&format=report&sort=date&format=&citing_topics=Statistics) | 1,096 |
| [Disease](https://app.overton.io/articles.php?identifiers=set%3A392%3A0f5c3195569f8372a21a47ce977169e8&format=report&sort=date&format=&citing_topics=Disease) | 1,080 |
| [Policy](https://app.overton.io/articles.php?identifiers=set%3A392%3A0f5c3195569f8372a21a47ce977169e8&format=report&sort=date&format=&citing_topics=Policy) | 1,077 |
| [Maternal health](https://app.overton.io/articles.php?identifiers=set%3A392%3A0f5c3195569f8372a21a47ce977169e8&format=report&sort=date&format=&citing_topics=Maternal%20health) | 1,073 |
| [Survey methodology](https://app.overton.io/articles.php?identifiers=set%3A392%3A0f5c3195569f8372a21a47ce977169e8&format=report&sort=date&format=&citing_topics=Survey%20methodology) | 1,043 |
| [Developing country](https://app.overton.io/articles.php?identifiers=set%3A392%3A0f5c3195569f8372a21a47ce977169e8&format=report&sort=date&format=&citing_topics=Developing%20country) | 1,035 |
| [Economic growth](https://app.overton.io/articles.php?identifiers=set%3A392%3A0f5c3195569f8372a21a47ce977169e8&format=report&sort=date&format=&citing_topics=Economic%20growth) | 1,029 |
| [Social issues](https://app.overton.io/articles.php?identifiers=set%3A392%3A0f5c3195569f8372a21a47ce977169e8&format=report&sort=date&format=&citing_topics=Social%20issues) | 1,014 |
| [Adolescence](https://app.overton.io/articles.php?identifiers=set%3A392%3A0f5c3195569f8372a21a47ce977169e8&format=report&sort=date&format=&citing_topics=Adolescence) | 1,005 |
| [HIV/AIDS](https://app.overton.io/articles.php?identifiers=set%3A392%3A0f5c3195569f8372a21a47ce977169e8&format=report&sort=date&format=&citing_topics=HIV%2FAIDS) | 982 |
| [Stunted growth](https://app.overton.io/articles.php?identifiers=set%3A392%3A0f5c3195569f8372a21a47ce977169e8&format=report&sort=date&format=&citing_topics=Stunted%20growth) | 977 |
| [Economic inequality](https://app.overton.io/articles.php?identifiers=set%3A392%3A0f5c3195569f8372a21a47ce977169e8&format=report&sort=date&format=&citing_topics=Economic%20inequality) | 972 |
| [Employment](https://app.overton.io/articles.php?identifiers=set%3A392%3A0f5c3195569f8372a21a47ce977169e8&format=report&sort=date&format=&citing_topics=Employment) | 965 |
| [Birth control](https://app.overton.io/articles.php?identifiers=set%3A392%3A0f5c3195569f8372a21a47ce977169e8&format=report&sort=date&format=&citing_topics=Birth%20control) | 929 |
| [Infant mortality](https://app.overton.io/articles.php?identifiers=set%3A392%3A0f5c3195569f8372a21a47ce977169e8&format=report&sort=date&format=&citing_topics=Infant%20mortality) | 927 |
| [Culture](https://app.overton.io/articles.php?identifiers=set%3A392%3A0f5c3195569f8372a21a47ce977169e8&format=report&sort=date&format=&citing_topics=Culture) | 910 |
| [Human reproduction](https://app.overton.io/articles.php?identifiers=set%3A392%3A0f5c3195569f8372a21a47ce977169e8&format=report&sort=date&format=&citing_topics=Human%20reproduction) | 907 |
| [Marriage](https://app.overton.io/articles.php?identifiers=set%3A392%3A0f5c3195569f8372a21a47ce977169e8&format=report&sort=date&format=&citing_topics=Marriage) | 904 |
| [Food security](https://app.overton.io/articles.php?identifiers=set%3A392%3A0f5c3195569f8372a21a47ce977169e8&format=report&sort=date&format=&citing_topics=Food%20security) | 894 |
| [Economics](https://app.overton.io/articles.php?identifiers=set%3A392%3A0f5c3195569f8372a21a47ce977169e8&format=report&sort=date&format=&citing_topics=Economics) | 878 |
| [Africa](https://app.overton.io/articles.php?identifiers=set%3A392%3A0f5c3195569f8372a21a47ce977169e8&format=report&sort=date&format=&citing_topics=Africa) | 874 |

**Supplementary Table S4: Classifications of citing policy**

| **Name** | **Articles** |
| --- | --- |
| [health](https://app.overton.io/articles.php?identifiers=set%3A392%3A0f5c3195569f8372a21a47ce977169e8&format=report&sort=date&format=&citing_classifications=health) | 2,902 |
| [science and technology](https://app.overton.io/articles.php?identifiers=set%3A392%3A0f5c3195569f8372a21a47ce977169e8&format=report&sort=date&format=&citing_classifications=science%20and%20technology) | 2,757 |
| [education](https://app.overton.io/articles.php?identifiers=set%3A392%3A0f5c3195569f8372a21a47ce977169e8&format=report&sort=date&format=&citing_classifications=education) | 2,109 |
| [health>diseases and conditions](https://app.overton.io/articles.php?identifiers=set%3A392%3A0f5c3195569f8372a21a47ce977169e8&format=report&sort=date&format=&citing_classifications=health%3Ediseases%20and%20conditions) | 1,916 |
| [science and technology>social sciences](https://app.overton.io/articles.php?identifiers=set%3A392%3A0f5c3195569f8372a21a47ce977169e8&format=report&sort=date&format=&citing_classifications=science%20and%20technology%3Esocial%20sciences) | 1,372 |
| [health>health treatment>medicine](https://app.overton.io/articles.php?identifiers=set%3A392%3A0f5c3195569f8372a21a47ce977169e8&format=report&sort=date&format=&citing_classifications=health%3Ehealth%20treatment%3Emedicine) | 1,144 |
| [science and technology>social sciences>economics](https://app.overton.io/articles.php?identifiers=set%3A392%3A0f5c3195569f8372a21a47ce977169e8&format=report&sort=date&format=&citing_classifications=science%20and%20technology%3Esocial%20sciences%3Eeconomics) | 1,029 |
| [society](https://app.overton.io/articles.php?identifiers=set%3A392%3A0f5c3195569f8372a21a47ce977169e8&format=report&sort=date&format=&citing_classifications=society) | 973 |
| [politics](https://app.overton.io/articles.php?identifiers=set%3A392%3A0f5c3195569f8372a21a47ce977169e8&format=report&sort=date&format=&citing_classifications=politics) | 914 |
| [education>school>further education](https://app.overton.io/articles.php?identifiers=set%3A392%3A0f5c3195569f8372a21a47ce977169e8&format=report&sort=date&format=&citing_classifications=education%3Eschool%3Efurther%20education) | 793 |
| [education>school](https://app.overton.io/articles.php?identifiers=set%3A392%3A0f5c3195569f8372a21a47ce977169e8&format=report&sort=date&format=&citing_classifications=education%3Eschool) | 735 |
| [economy, business and finance>economic sector>consumer goods>food](https://app.overton.io/articles.php?identifiers=set%3A392%3A0f5c3195569f8372a21a47ce977169e8&format=report&sort=date&format=&citing_classifications=economy%2C%20business%20and%20finance%3Eeconomic%20sector%3Econsumer%20goods%3Efood) | 673 |
| [environment](https://app.overton.io/articles.php?identifiers=set%3A392%3A0f5c3195569f8372a21a47ce977169e8&format=report&sort=date&format=&citing_classifications=environment) | 661 |
| [science and technology>mathematics](https://app.overton.io/articles.php?identifiers=set%3A392%3A0f5c3195569f8372a21a47ce977169e8&format=report&sort=date&format=&citing_classifications=science%20and%20technology%3Emathematics) | 628 |
| [lifestyle and leisure>lifestyle>food and drink](https://app.overton.io/articles.php?identifiers=set%3A392%3A0f5c3195569f8372a21a47ce977169e8&format=report&sort=date&format=&citing_classifications=lifestyle%20and%20leisure%3Elifestyle%3Efood%20and%20drink) | 616 |
| [health>diseases and conditions>communicable disease>virus disease](https://app.overton.io/articles.php?identifiers=set%3A392%3A0f5c3195569f8372a21a47ce977169e8&format=report&sort=date&format=&citing_classifications=health%3Ediseases%20and%20conditions%3Ecommunicable%20disease%3Evirus%20disease) | 591 |
| [economy, business and finance>economy](https://app.overton.io/articles.php?identifiers=set%3A392%3A0f5c3195569f8372a21a47ce977169e8&format=report&sort=date&format=&citing_classifications=economy%2C%20business%20and%20finance%3Eeconomy) | 571 |
| [health>health treatment](https://app.overton.io/articles.php?identifiers=set%3A392%3A0f5c3195569f8372a21a47ce977169e8&format=report&sort=date&format=&citing_classifications=health%3Ehealth%20treatment) | 539 |
| [health>diseases and conditions>illness](https://app.overton.io/articles.php?identifiers=set%3A392%3A0f5c3195569f8372a21a47ce977169e8&format=report&sort=date&format=&citing_classifications=health%3Ediseases%20and%20conditions%3Eillness) | 512 |
| [society>family](https://app.overton.io/articles.php?identifiers=set%3A392%3A0f5c3195569f8372a21a47ce977169e8&format=report&sort=date&format=&citing_classifications=society%3Efamily) | 506 |
| [science and technology>social sciences>psychology](https://app.overton.io/articles.php?identifiers=set%3A392%3A0f5c3195569f8372a21a47ce977169e8&format=report&sort=date&format=&citing_classifications=science%20and%20technology%3Esocial%20sciences%3Epsychology) | 502 |
| [health>health treatment>diet](https://app.overton.io/articles.php?identifiers=set%3A392%3A0f5c3195569f8372a21a47ce977169e8&format=report&sort=date&format=&citing_classifications=health%3Ehealth%20treatment%3Ediet) | 500 |
| [labour](https://app.overton.io/articles.php?identifiers=set%3A392%3A0f5c3195569f8372a21a47ce977169e8&format=report&sort=date&format=&citing_classifications=labour) | 427 |
| [politics>government](https://app.overton.io/articles.php?identifiers=set%3A392%3A0f5c3195569f8372a21a47ce977169e8&format=report&sort=date&format=&citing_classifications=politics%3Egovernment) | 419 |
| [health>diseases and conditions>communicable disease>virus disease>AIDS](https://app.overton.io/articles.php?identifiers=set%3A392%3A0f5c3195569f8372a21a47ce977169e8&format=report&sort=date&format=&citing_classifications=health%3Ediseases%20and%20conditions%3Ecommunicable%20disease%3Evirus%20disease%3EAIDS) | 386 |
| [economy, business and finance>economic sector>agriculture](https://app.overton.io/articles.php?identifiers=set%3A392%3A0f5c3195569f8372a21a47ce977169e8&format=report&sort=date&format=&citing_classifications=economy%2C%20business%20and%20finance%3Eeconomic%20sector%3Eagriculture) | 372 |
| [health>diseases and conditions>communicable disease](https://app.overton.io/articles.php?identifiers=set%3A392%3A0f5c3195569f8372a21a47ce977169e8&format=report&sort=date&format=&citing_classifications=health%3Ediseases%20and%20conditions%3Ecommunicable%20disease) | 319 |
| [society>social condition>poverty](https://app.overton.io/articles.php?identifiers=set%3A392%3A0f5c3195569f8372a21a47ce977169e8&format=report&sort=date&format=&citing_classifications=society%3Esocial%20condition%3Epoverty) | 310 |
| [science and technology>natural science>biology](https://app.overton.io/articles.php?identifiers=set%3A392%3A0f5c3195569f8372a21a47ce977169e8&format=report&sort=date&format=&citing_classifications=science%20and%20technology%3Enatural%20science%3Ebiology) | 283 |
| [society>values](https://app.overton.io/articles.php?identifiers=set%3A392%3A0f5c3195569f8372a21a47ce977169e8&format=report&sort=date&format=&citing_classifications=society%3Evalues) | 263 |
| [society>family>marriage](https://app.overton.io/articles.php?identifiers=set%3A392%3A0f5c3195569f8372a21a47ce977169e8&format=report&sort=date&format=&citing_classifications=society%3Efamily%3Emarriage) | 261 |
| [economy, business and finance>economic sector](https://app.overton.io/articles.php?identifiers=set%3A392%3A0f5c3195569f8372a21a47ce977169e8&format=report&sort=date&format=&citing_classifications=economy%2C%20business%20and%20finance%3Eeconomic%20sector) | 244 |
| [environment>natural resources>water](https://app.overton.io/articles.php?identifiers=set%3A392%3A0f5c3195569f8372a21a47ce977169e8&format=report&sort=date&format=&citing_classifications=environment%3Enatural%20resources%3Ewater) | 222 |
| [society>values>ethics](https://app.overton.io/articles.php?identifiers=set%3A392%3A0f5c3195569f8372a21a47ce977169e8&format=report&sort=date&format=&citing_classifications=society%3Evalues%3Eethics) | 197 |
| [environment>environmental pollution](https://app.overton.io/articles.php?identifiers=set%3A392%3A0f5c3195569f8372a21a47ce977169e8&format=report&sort=date&format=&citing_classifications=environment%3Eenvironmental%20pollution) | 187 |
| [crime, law and justice>crime](https://app.overton.io/articles.php?identifiers=set%3A392%3A0f5c3195569f8372a21a47ce977169e8&format=report&sort=date&format=&citing_classifications=crime%2C%20law%20and%20justice%3Ecrime) | 173 |
| [health>diseases and conditions>cancer](https://app.overton.io/articles.php?identifiers=set%3A392%3A0f5c3195569f8372a21a47ce977169e8&format=report&sort=date&format=&citing_classifications=health%3Ediseases%20and%20conditions%3Ecancer) | 172 |
| [economy, business and finance>economic sector>computing and information technology](https://app.overton.io/articles.php?identifiers=set%3A392%3A0f5c3195569f8372a21a47ce977169e8&format=report&sort=date&format=&citing_classifications=economy%2C%20business%20and%20finance%3Eeconomic%20sector%3Ecomputing%20and%20information%20technology) | 168 |
| [society>family>family planning](https://app.overton.io/articles.php?identifiers=set%3A392%3A0f5c3195569f8372a21a47ce977169e8&format=report&sort=date&format=&citing_classifications=society%3Efamily%3Efamily%20planning) | 161 |
| [economy, business and finance>economic sector>energy and resource](https://app.overton.io/articles.php?identifiers=set%3A392%3A0f5c3195569f8372a21a47ce977169e8&format=report&sort=date&format=&citing_classifications=economy%2C%20business%20and%20finance%3Eeconomic%20sector%3Eenergy%20and%20resource) | 160 |
| [disaster, accident and emergency incident>disaster](https://app.overton.io/articles.php?identifiers=set%3A392%3A0f5c3195569f8372a21a47ce977169e8&format=report&sort=date&format=&citing_classifications=disaster%2C%20accident%20and%20emergency%20incident%3Edisaster) | 159 |
| [conflicts, war and peace](https://app.overton.io/articles.php?identifiers=set%3A392%3A0f5c3195569f8372a21a47ce977169e8&format=report&sort=date&format=&citing_classifications=conflicts%2C%20war%20and%20peace) | 152 |
| [education>school>higher education>university](https://app.overton.io/articles.php?identifiers=set%3A392%3A0f5c3195569f8372a21a47ce977169e8&format=report&sort=date&format=&citing_classifications=education%3Eschool%3Ehigher%20education%3Euniversity) | 148 |
| [society>social condition](https://app.overton.io/articles.php?identifiers=set%3A392%3A0f5c3195569f8372a21a47ce977169e8&format=report&sort=date&format=&citing_classifications=society%3Esocial%20condition) | 148 |
| [environment>natural resources](https://app.overton.io/articles.php?identifiers=set%3A392%3A0f5c3195569f8372a21a47ce977169e8&format=report&sort=date&format=&citing_classifications=environment%3Enatural%20resources) | 136 |
| [society>discrimination](https://app.overton.io/articles.php?identifiers=set%3A392%3A0f5c3195569f8372a21a47ce977169e8&format=report&sort=date&format=&citing_classifications=society%3Ediscrimination) | 136 |
| [labour>unemployment](https://app.overton.io/articles.php?identifiers=set%3A392%3A0f5c3195569f8372a21a47ce977169e8&format=report&sort=date&format=&citing_classifications=labour%3Eunemployment) | 132 |
| [health>diseases and conditions>obesity](https://app.overton.io/articles.php?identifiers=set%3A392%3A0f5c3195569f8372a21a47ce977169e8&format=report&sort=date&format=&citing_classifications=health%3Ediseases%20and%20conditions%3Eobesity) | 131 |
| [health>health treatment>preventative medicine](https://app.overton.io/articles.php?identifiers=set%3A392%3A0f5c3195569f8372a21a47ce977169e8&format=report&sort=date&format=&citing_classifications=health%3Ehealth%20treatment%3Epreventative%20medicine) | 123 |
| [labour>employment](https://app.overton.io/articles.php?identifiers=set%3A392%3A0f5c3195569f8372a21a47ce977169e8&format=report&sort=date&format=&citing_classifications=labour%3Eemployment) | 122 |

**Supplementary Table S5: Journal subjects**

| **Name** | **Articles** |
| --- | --- |
| [Public Health, Environmental and Occupational Health](https://app.overton.io/articles.php?identifiers=set%3A392%3A0f5c3195569f8372a21a47ce977169e8&format=report&sort=date&format=&with_journal_subject=Public%20Health%2C%20Environmental%20and%20Occupational%20Health) | 901 |
| [Demography](https://app.overton.io/articles.php?identifiers=set%3A392%3A0f5c3195569f8372a21a47ce977169e8&format=report&sort=date&format=&with_journal_subject=Demography) | 338 |
| [Health Policy](https://app.overton.io/articles.php?identifiers=set%3A392%3A0f5c3195569f8372a21a47ce977169e8&format=report&sort=date&format=&with_journal_subject=Health%20Policy) | 283 |
| [Obstetrics and Gynecology](https://app.overton.io/articles.php?identifiers=set%3A392%3A0f5c3195569f8372a21a47ce977169e8&format=report&sort=date&format=&with_journal_subject=Obstetrics%20and%20Gynecology) | 278 |
| [Biochemistry, Genetics and Molecular Biology (all)](https://app.overton.io/articles.php?identifiers=set%3A392%3A0f5c3195569f8372a21a47ce977169e8&format=report&sort=date&format=&with_journal_subject=Biochemistry%2C%20Genetics%20and%20Molecular%20Biology%20%28all%29) | 257 |
| [Multidisciplinary](https://app.overton.io/articles.php?identifiers=set%3A392%3A0f5c3195569f8372a21a47ce977169e8&format=report&sort=date&format=&with_journal_subject=Multidisciplinary) | 256 |
| [Agricultural and Biological Sciences (all)](https://app.overton.io/articles.php?identifiers=set%3A392%3A0f5c3195569f8372a21a47ce977169e8&format=report&sort=date&format=&with_journal_subject=Agricultural%20and%20Biological%20Sciences%20%28all%29) | 237 |
| [Development](https://app.overton.io/articles.php?identifiers=set%3A392%3A0f5c3195569f8372a21a47ce977169e8&format=report&sort=date&format=&with_journal_subject=Development) | 181 |
| [Medicine (all)](https://app.overton.io/articles.php?identifiers=set%3A392%3A0f5c3195569f8372a21a47ce977169e8&format=report&sort=date&format=&with_journal_subject=Medicine%20%28all%29) | 180 |
| [Health (social science)](https://app.overton.io/articles.php?identifiers=set%3A392%3A0f5c3195569f8372a21a47ce977169e8&format=report&sort=date&format=&with_journal_subject=Health%20%28social%20science%29) | 178 |
| [Sociology and Political Science](https://app.overton.io/articles.php?identifiers=set%3A392%3A0f5c3195569f8372a21a47ce977169e8&format=report&sort=date&format=&with_journal_subject=Sociology%20and%20Political%20Science) | 178 |
| [Geography, Planning and Development](https://app.overton.io/articles.php?identifiers=set%3A392%3A0f5c3195569f8372a21a47ce977169e8&format=report&sort=date&format=&with_journal_subject=Geography%2C%20Planning%20and%20Development) | 176 |
| [Nutrition and Dietetics](https://app.overton.io/articles.php?identifiers=set%3A392%3A0f5c3195569f8372a21a47ce977169e8&format=report&sort=date&format=&with_journal_subject=Nutrition%20and%20Dietetics) | 160 |
| [Economics and Econometrics](https://app.overton.io/articles.php?identifiers=set%3A392%3A0f5c3195569f8372a21a47ce977169e8&format=report&sort=date&format=&with_journal_subject=Economics%20and%20Econometrics) | 157 |
| [Infectious Diseases](https://app.overton.io/articles.php?identifiers=set%3A392%3A0f5c3195569f8372a21a47ce977169e8&format=report&sort=date&format=&with_journal_subject=Infectious%20Diseases) | 149 |
| [Pediatrics, Perinatology and Child Health](https://app.overton.io/articles.php?identifiers=set%3A392%3A0f5c3195569f8372a21a47ce977169e8&format=report&sort=date&format=&with_journal_subject=Pediatrics%2C%20Perinatology%20and%20Child%20Health) | 124 |
| [Social Sciences (all)](https://app.overton.io/articles.php?identifiers=set%3A392%3A0f5c3195569f8372a21a47ce977169e8&format=report&sort=date&format=&with_journal_subject=Social%20Sciences%20%28all%29) | 123 |
| [Social Sciences (miscellaneous)](https://app.overton.io/articles.php?identifiers=set%3A392%3A0f5c3195569f8372a21a47ce977169e8&format=report&sort=date&format=&with_journal_subject=Social%20Sciences%20%28miscellaneous%29) | 122 |
| [Reproductive Medicine](https://app.overton.io/articles.php?identifiers=set%3A392%3A0f5c3195569f8372a21a47ce977169e8&format=report&sort=date&format=&with_journal_subject=Reproductive%20Medicine) | 116 |
| [Epidemiology](https://app.overton.io/articles.php?identifiers=set%3A392%3A0f5c3195569f8372a21a47ce977169e8&format=report&sort=date&format=&with_journal_subject=Epidemiology) | 112 |
| [Medicine (miscellaneous)](https://app.overton.io/articles.php?identifiers=set%3A392%3A0f5c3195569f8372a21a47ce977169e8&format=report&sort=date&format=&with_journal_subject=Medicine%20%28miscellaneous%29) | 106 |
| [History and Philosophy of Science](https://app.overton.io/articles.php?identifiers=set%3A392%3A0f5c3195569f8372a21a47ce977169e8&format=report&sort=date&format=&with_journal_subject=History%20and%20Philosophy%20of%20Science) | 73 |
| [Food Science](https://app.overton.io/articles.php?identifiers=set%3A392%3A0f5c3195569f8372a21a47ce977169e8&format=report&sort=date&format=&with_journal_subject=Food%20Science) | 57 |
| [Management, Monitoring, Policy and Law](https://app.overton.io/articles.php?identifiers=set%3A392%3A0f5c3195569f8372a21a47ce977169e8&format=report&sort=date&format=&with_journal_subject=Management%2C%20Monitoring%2C%20Policy%20and%20Law) | 46 |
| [Arts and Humanities (miscellaneous)](https://app.overton.io/articles.php?identifiers=set%3A392%3A0f5c3195569f8372a21a47ce977169e8&format=report&sort=date&format=&with_journal_subject=Arts%20and%20Humanities%20%28miscellaneous%29) | 45 |
| [Health, Toxicology and Mutagenesis](https://app.overton.io/articles.php?identifiers=set%3A392%3A0f5c3195569f8372a21a47ce977169e8&format=report&sort=date&format=&with_journal_subject=Health%2C%20Toxicology%20and%20Mutagenesis) | 41 |
| [History](https://app.overton.io/articles.php?identifiers=set%3A392%3A0f5c3195569f8372a21a47ce977169e8&format=report&sort=date&format=&with_journal_subject=History) | 41 |
| [Parasitology](https://app.overton.io/articles.php?identifiers=set%3A392%3A0f5c3195569f8372a21a47ce977169e8&format=report&sort=date&format=&with_journal_subject=Parasitology) | 39 |
| [Clinical Psychology](https://app.overton.io/articles.php?identifiers=set%3A392%3A0f5c3195569f8372a21a47ce977169e8&format=report&sort=date&format=&with_journal_subject=Clinical%20Psychology) | 38 |
| [Social Psychology](https://app.overton.io/articles.php?identifiers=set%3A392%3A0f5c3195569f8372a21a47ce977169e8&format=report&sort=date&format=&with_journal_subject=Social%20Psychology) | 38 |
| [Anthropology](https://app.overton.io/articles.php?identifiers=set%3A392%3A0f5c3195569f8372a21a47ce977169e8&format=report&sort=date&format=&with_journal_subject=Anthropology) | 33 |
| [Education](https://app.overton.io/articles.php?identifiers=set%3A392%3A0f5c3195569f8372a21a47ce977169e8&format=report&sort=date&format=&with_journal_subject=Education) | 31 |
| [Applied Psychology](https://app.overton.io/articles.php?identifiers=set%3A392%3A0f5c3195569f8372a21a47ce977169e8&format=report&sort=date&format=&with_journal_subject=Applied%20Psychology) | 28 |
| [Developmental and Educational Psychology](https://app.overton.io/articles.php?identifiers=set%3A392%3A0f5c3195569f8372a21a47ce977169e8&format=report&sort=date&format=&with_journal_subject=Developmental%20and%20Educational%20Psychology) | 27 |
| [Immunology and Microbiology (all)](https://app.overton.io/articles.php?identifiers=set%3A392%3A0f5c3195569f8372a21a47ce977169e8&format=report&sort=date&format=&with_journal_subject=Immunology%20and%20Microbiology%20%28all%29) | 25 |
| [Immunology](https://app.overton.io/articles.php?identifiers=set%3A392%3A0f5c3195569f8372a21a47ce977169e8&format=report&sort=date&format=&with_journal_subject=Immunology) | 21 |
| [Dermatology](https://app.overton.io/articles.php?identifiers=set%3A392%3A0f5c3195569f8372a21a47ce977169e8&format=report&sort=date&format=&with_journal_subject=Dermatology) | 20 |
| [Economics, Econometrics and Finance (miscellaneous)](https://app.overton.io/articles.php?identifiers=set%3A392%3A0f5c3195569f8372a21a47ce977169e8&format=report&sort=date&format=&with_journal_subject=Economics%2C%20Econometrics%20and%20Finance%20%28miscellaneous%29) | 20 |
| [Virology](https://app.overton.io/articles.php?identifiers=set%3A392%3A0f5c3195569f8372a21a47ce977169e8&format=report&sort=date&format=&with_journal_subject=Virology) | 20 |
| [Gender Studies](https://app.overton.io/articles.php?identifiers=set%3A392%3A0f5c3195569f8372a21a47ce977169e8&format=report&sort=date&format=&with_journal_subject=Gender%20Studies) | 18 |
| [Psychiatry and Mental Health](https://app.overton.io/articles.php?identifiers=set%3A392%3A0f5c3195569f8372a21a47ce977169e8&format=report&sort=date&format=&with_journal_subject=Psychiatry%20and%20Mental%20Health) | 17 |
| [Ecology, Evolution, Behavior and Systematics](https://app.overton.io/articles.php?identifiers=set%3A392%3A0f5c3195569f8372a21a47ce977169e8&format=report&sort=date&format=&with_journal_subject=Ecology%2C%20Evolution%2C%20Behavior%20and%20Systematics) | 16 |
| [Environmental Science (miscellaneous)](https://app.overton.io/articles.php?identifiers=set%3A392%3A0f5c3195569f8372a21a47ce977169e8&format=report&sort=date&format=&with_journal_subject=Environmental%20Science%20%28miscellaneous%29) | 16 |
| [Finance](https://app.overton.io/articles.php?identifiers=set%3A392%3A0f5c3195569f8372a21a47ce977169e8&format=report&sort=date&format=&with_journal_subject=Finance) | 16 |
| [Immunology and Allergy](https://app.overton.io/articles.php?identifiers=set%3A392%3A0f5c3195569f8372a21a47ce977169e8&format=report&sort=date&format=&with_journal_subject=Immunology%20and%20Allergy) | 16 |
| [Business, Management and Accounting (all)](https://app.overton.io/articles.php?identifiers=set%3A392%3A0f5c3195569f8372a21a47ce977169e8&format=report&sort=date&format=&with_journal_subject=Business%2C%20Management%20and%20Accounting%20%28all%29) | 15 |
| [Genetics](https://app.overton.io/articles.php?identifiers=set%3A392%3A0f5c3195569f8372a21a47ce977169e8&format=report&sort=date&format=&with_journal_subject=Genetics) | 15 |
| [Maternity and Midwifery](https://app.overton.io/articles.php?identifiers=set%3A392%3A0f5c3195569f8372a21a47ce977169e8&format=report&sort=date&format=&with_journal_subject=Maternity%20and%20Midwifery) | 15 |
| [Statistics and Probability](https://app.overton.io/articles.php?identifiers=set%3A392%3A0f5c3195569f8372a21a47ce977169e8&format=report&sort=date&format=&with_journal_subject=Statistics%20and%20Probability) | 15 |
| [Law](https://app.overton.io/articles.php?identifiers=set%3A392%3A0f5c3195569f8372a21a47ce977169e8&format=report&sort=date&format=&with_journal_subject=Law) | 14 |

**Supplementary Table S6: Author affiliations**

| **Name** | **Articles** |
| --- | --- |
| [Imperial College London](https://app.overton.io/articles.php?identifiers=set%3A392%3A0f5c3195569f8372a21a47ce977169e8&format=report&sort=date&format=&open_affiliations=Imperial%20College%20London) | 34 |
| [Johns Hopkins University](https://app.overton.io/articles.php?identifiers=set%3A392%3A0f5c3195569f8372a21a47ce977169e8&format=report&sort=date&format=&open_affiliations=Johns%20Hopkins%20University) | 145 |
| [London School of Hygiene & Tropical Medicine](https://app.overton.io/articles.php?identifiers=set%3A392%3A0f5c3195569f8372a21a47ce977169e8&format=report&sort=date&format=&open_affiliations=London%20School%20of%20Hygiene%20%26%20Tropical%20Medicine) | 134 |
| [Harvard University](https://app.overton.io/articles.php?identifiers=set%3A392%3A0f5c3195569f8372a21a47ce977169e8&format=report&sort=date&format=&open_affiliations=Harvard%20University) | 112 |
| [World Health Organization - Pakistan](https://app.overton.io/articles.php?identifiers=set%3A392%3A0f5c3195569f8372a21a47ce977169e8&format=report&sort=date&format=&open_affiliations=World%20Health%20Organization%20-%20Pakistan) | 82 |
| [University of Cape Coast](https://app.overton.io/articles.php?identifiers=set%3A392%3A0f5c3195569f8372a21a47ce977169e8&format=report&sort=date&format=&open_affiliations=University%20of%20Cape%20Coast) | 70 |
| [University of North Carolina at Chapel Hill](https://app.overton.io/articles.php?identifiers=set%3A392%3A0f5c3195569f8372a21a47ce977169e8&format=report&sort=date&format=&open_affiliations=University%20of%20North%20Carolina%20at%20Chapel%20Hill) | 69 |
| [University of Southampton](https://app.overton.io/articles.php?identifiers=set%3A392%3A0f5c3195569f8372a21a47ce977169e8&format=report&sort=date&format=&open_affiliations=University%20of%20Southampton) | 67 |
| [African Population and Health Research Center](https://app.overton.io/articles.php?identifiers=set%3A392%3A0f5c3195569f8372a21a47ce977169e8&format=report&sort=date&format=&open_affiliations=African%20Population%20and%20Health%20Research%20Center) | 64 |
| [Harvard Global Health Institute](https://app.overton.io/articles.php?identifiers=set%3A392%3A0f5c3195569f8372a21a47ce977169e8&format=report&sort=date&format=&open_affiliations=Harvard%20Global%20Health%20Institute) | 64 |
| [University Of Gondar](https://app.overton.io/articles.php?identifiers=set%3A392%3A0f5c3195569f8372a21a47ce977169e8&format=report&sort=date&format=&open_affiliations=University%20Of%20Gondar) | 64 |
| [University of Technology Sydney](https://app.overton.io/articles.php?identifiers=set%3A392%3A0f5c3195569f8372a21a47ce977169e8&format=report&sort=date&format=&open_affiliations=University%20of%20Technology%20Sydney) | 59 |
| [University of Sydney](https://app.overton.io/articles.php?identifiers=set%3A392%3A0f5c3195569f8372a21a47ce977169e8&format=report&sort=date&format=&open_affiliations=University%20of%20Sydney) | 57 |
| [United Nations Children's Fund](https://app.overton.io/articles.php?identifiers=set%3A392%3A0f5c3195569f8372a21a47ce977169e8&format=report&sort=date&format=&open_affiliations=United%20Nations%20Children%27s%20Fund) | 55 |
| [University of Ottawa](https://app.overton.io/articles.php?identifiers=set%3A392%3A0f5c3195569f8372a21a47ce977169e8&format=report&sort=date&format=&open_affiliations=University%20of%20Ottawa) | 53 |
| [World Bank](https://app.overton.io/articles.php?identifiers=set%3A392%3A0f5c3195569f8372a21a47ce977169e8&format=report&sort=date&format=&open_affiliations=World%20Bank) | 53 |
| [Universidade Federal de Pelotas](https://app.overton.io/articles.php?identifiers=set%3A392%3A0f5c3195569f8372a21a47ce977169e8&format=report&sort=date&format=&open_affiliations=Universidade%20Federal%20de%20Pelotas) | 52 |
| [University of the Witwatersrand](https://app.overton.io/articles.php?identifiers=set%3A392%3A0f5c3195569f8372a21a47ce977169e8&format=report&sort=date&format=&open_affiliations=University%20of%20the%20Witwatersrand) | 51 |
| [Western Sydney University](https://app.overton.io/articles.php?identifiers=set%3A392%3A0f5c3195569f8372a21a47ce977169e8&format=report&sort=date&format=&open_affiliations=Western%20Sydney%20University) | 51 |
| [University of Ghana](https://app.overton.io/articles.php?identifiers=set%3A392%3A0f5c3195569f8372a21a47ce977169e8&format=report&sort=date&format=&open_affiliations=University%20of%20Ghana) | 49 |
| [International Centre for Diarrhoeal Disease Research](https://app.overton.io/articles.php?identifiers=set%3A392%3A0f5c3195569f8372a21a47ce977169e8&format=report&sort=date&format=&open_affiliations=International%20Centre%20for%20Diarrhoeal%20Disease%20Research) | 44 |
| [Karolinska Institute](https://app.overton.io/articles.php?identifiers=set%3A392%3A0f5c3195569f8372a21a47ce977169e8&format=report&sort=date&format=&open_affiliations=Karolinska%20Institute) | 44 |
| [Emory University](https://app.overton.io/articles.php?identifiers=set%3A392%3A0f5c3195569f8372a21a47ce977169e8&format=report&sort=date&format=&open_affiliations=Emory%20University) | 43 |
| [Makerere University](https://app.overton.io/articles.php?identifiers=set%3A392%3A0f5c3195569f8372a21a47ce977169e8&format=report&sort=date&format=&open_affiliations=Makerere%20University) | 43 |
| [Pennsylvania State University](https://app.overton.io/articles.php?identifiers=set%3A392%3A0f5c3195569f8372a21a47ce977169e8&format=report&sort=date&format=&open_affiliations=Pennsylvania%20State%20University) | 43 |
| [Tulane University](https://app.overton.io/articles.php?identifiers=set%3A392%3A0f5c3195569f8372a21a47ce977169e8&format=report&sort=date&format=&open_affiliations=Tulane%20University) | 43 |
| [University of Ibadan](https://app.overton.io/articles.php?identifiers=set%3A392%3A0f5c3195569f8372a21a47ce977169e8&format=report&sort=date&format=&open_affiliations=University%20of%20Ibadan) | 41 |
| [Addis Ababa University](https://app.overton.io/articles.php?identifiers=set%3A392%3A0f5c3195569f8372a21a47ce977169e8&format=report&sort=date&format=&open_affiliations=Addis%20Ababa%20University) | 39 |
| [ICF International (United States)](https://app.overton.io/articles.php?identifiers=set%3A392%3A0f5c3195569f8372a21a47ce977169e8&format=report&sort=date&format=&open_affiliations=ICF%20International%20%28United%20States%29) | 36 |
| [International Food Policy Research Institute](https://app.overton.io/articles.php?identifiers=set%3A392%3A0f5c3195569f8372a21a47ce977169e8&format=report&sort=date&format=&open_affiliations=International%20Food%20Policy%20Research%20Institute) | 36 |
| [Population Council](https://app.overton.io/articles.php?identifiers=set%3A392%3A0f5c3195569f8372a21a47ce977169e8&format=report&sort=date&format=&open_affiliations=Population%20Council) | 35 |
| [University of Michigan–Ann Arbor](https://app.overton.io/articles.php?identifiers=set%3A392%3A0f5c3195569f8372a21a47ce977169e8&format=report&sort=date&format=&open_affiliations=University%20of%20Michigan%E2%80%93Ann%20Arbor) | 35 |
| [Ministry of Health](https://app.overton.io/articles.php?identifiers=set%3A392%3A0f5c3195569f8372a21a47ce977169e8&format=report&sort=date&format=&open_affiliations=Ministry%20of%20Health) | 34 |
| [University of Oxford](https://app.overton.io/articles.php?identifiers=set%3A392%3A0f5c3195569f8372a21a47ce977169e8&format=report&sort=date&format=&open_affiliations=University%20of%20Oxford) | 34 |
| [McGill University](https://app.overton.io/articles.php?identifiers=set%3A392%3A0f5c3195569f8372a21a47ce977169e8&format=report&sort=date&format=&open_affiliations=McGill%20University) | 33 |
| [University of Washington](https://app.overton.io/articles.php?identifiers=set%3A392%3A0f5c3195569f8372a21a47ce977169e8&format=report&sort=date&format=&open_affiliations=University%20of%20Washington) | 33 |
| [University of London](https://app.overton.io/articles.php?identifiers=set%3A392%3A0f5c3195569f8372a21a47ce977169e8&format=report&sort=date&format=&open_affiliations=University%20of%20London) | 31 |
| [University of Dhaka](https://app.overton.io/articles.php?identifiers=set%3A392%3A0f5c3195569f8372a21a47ce977169e8&format=report&sort=date&format=&open_affiliations=University%20of%20Dhaka) | 29 |
| [University of Queensland](https://app.overton.io/articles.php?identifiers=set%3A392%3A0f5c3195569f8372a21a47ce977169e8&format=report&sort=date&format=&open_affiliations=University%20of%20Queensland) | 28 |
| [James Cook University](https://app.overton.io/articles.php?identifiers=set%3A392%3A0f5c3195569f8372a21a47ce977169e8&format=report&sort=date&format=&open_affiliations=James%20Cook%20University) | 27 |
| [University College London](https://app.overton.io/articles.php?identifiers=set%3A392%3A0f5c3195569f8372a21a47ce977169e8&format=report&sort=date&format=&open_affiliations=University%20College%20London) | 27 |
| [University of KwaZulu-Natal](https://app.overton.io/articles.php?identifiers=set%3A392%3A0f5c3195569f8372a21a47ce977169e8&format=report&sort=date&format=&open_affiliations=University%20of%20KwaZulu-Natal) | 27 |
| [Stanford University](https://app.overton.io/articles.php?identifiers=set%3A392%3A0f5c3195569f8372a21a47ce977169e8&format=report&sort=date&format=&open_affiliations=Stanford%20University) | 26 |
| [University of Cape Town](https://app.overton.io/articles.php?identifiers=set%3A392%3A0f5c3195569f8372a21a47ce977169e8&format=report&sort=date&format=&open_affiliations=University%20of%20Cape%20Town) | 26 |
| [University of Warwick](https://app.overton.io/articles.php?identifiers=set%3A392%3A0f5c3195569f8372a21a47ce977169e8&format=report&sort=date&format=&open_affiliations=University%20of%20Warwick) | 26 |
| [University of Health and Allied Sciences](https://app.overton.io/articles.php?identifiers=set%3A392%3A0f5c3195569f8372a21a47ce977169e8&format=report&sort=date&format=&open_affiliations=University%20of%20Health%20and%20Allied%20Sciences) | 25 |
| [Obafemi Awolowo University](https://app.overton.io/articles.php?identifiers=set%3A392%3A0f5c3195569f8372a21a47ce977169e8&format=report&sort=date&format=&open_affiliations=Obafemi%20Awolowo%20University) | 24 |
| [United States Agency for International Development](https://app.overton.io/articles.php?identifiers=set%3A392%3A0f5c3195569f8372a21a47ce977169e8&format=report&sort=date&format=&open_affiliations=United%20States%20Agency%20for%20International%20Development) | 21 |
| [Bielefeld University](https://app.overton.io/articles.php?identifiers=set%3A392%3A0f5c3195569f8372a21a47ce977169e8&format=report&sort=date&format=&open_affiliations=Bielefeld%20University) | 20 |
| [University of California, Los Angeles](https://app.overton.io/articles.php?identifiers=set%3A392%3A0f5c3195569f8372a21a47ce977169e8&format=report&sort=date&format=&open_affiliations=University%20of%20California%2C%20Los%20Angeles) | 20 |
